# Epigenomics and bolting tolerance in sugar beet genotypes

**DOI:** 10.1093/jxb/erv449

**Published:** 2015-10-13

**Authors:** Claire Hébrard, Daniel G. Peterson, Glenda Willems, Alain Delaunay, Béline Jesson, Marc Lefèbvre, Steve Barnes, Stéphane Maury

**Affiliations:** ^1^Université d’Orléans, Faculté des Sciences, Laboratoire de Biologie des Ligneux et des Grandes Cultures (LBLGC), UPRES EA 1207, 45067 Orléans, France; ^2^INRA, USC1328 Arbres et Réponses aux Contraintes Hydriques et Environnementales (ARCHE), 45067 Orléans, France; ^3^SESVanderHave N.V./S.A., Soldatenplein Z2 nr15, Industriepark, B-3300 Tienen, Belgium; ^4^Institute for Genomics, Biocomputing & Biotechnology, Mississippi State University, 2 Research Blvd., Box 9627, Mississippi State, MS 39762, USA; ^5^IMAXIO/HELIXIO, Biopôle Clermont-Limagne, Saint-Beauzire, F-63360, France

**Keywords:** Bolting tolerance, differentially expressed gene, differentially methylated region, epigenomics, microarray, sugar beet, vernalization.

## Abstract

In sugar beet (*Beta vulgaris altissima*), bolting tolerance is an essential agronomic trait reflecting the bolting response of genotypes after vernalization. Genes involved in induction of sugar beet bolting have now been identified, and evidence suggests that epigenetic factors are involved in their control. Indeed, the time course and amplitude of DNA methylation variations in the shoot apical meristem have been shown to be critical in inducing sugar beet bolting, and a few functional targets of DNA methylation during vernalization have been identified. However, molecular mechanisms controlling bolting tolerance levels among genotypes are still poorly understood. Here, gene expression and DNA methylation profiles were compared in shoot apical meristems of three bolting-resistant and three bolting-sensitive genotypes after vernalization. Using Cot fractionation followed by 454 sequencing of the isolated low-copy DNA, 6231 contigs were obtained that were used along with public sugar beet DNA sequences to design custom Agilent microarrays for expression (56k) and methylation (244k) analyses. A total of 169 differentially expressed genes and 111 differentially methylated regions were identified between resistant and sensitive vernalized genotypes. Fourteen sequences were both differentially expressed and differentially methylated, with a negative correlation between their methylation and expression levels. Genes involved in cold perception, phytohormone signalling, and flowering induction were over-represented and collectively represent an integrative gene network from environmental perception to bolting induction. Altogether, the data suggest that the genotype-dependent control of DNA methylation and expression of an integrative gene network participate in bolting tolerance in sugar beet, opening up perspectives for crop improvement.

## Highlight

Sugar beet bolting tolerance is associated, in the shoot apical meristem, with genotype-dependent DNA methylation and expression variations of an integrative gene network involved in environmental perception and flowering.

## Introduction

Sugar beet (*Beta vulgaris altissima*) is a biennial root crop that provides ~15% of the world’s sugar (FAO, 2012). This species is generally grown as a spring-sown crop and harvested the following autumn, the whole initial growing season corresponding to vegetative growth and sucrose production and storage. To induce bolting (i.e. rapid stem elongation followed by flowering), sugar beet requires a prolonged exposure to low temperatures between 2 °C and 10 °C (Lexander, 1980), a phenomenon called vernalization. This process requires mitotic activity of somatic cells in the shoot apical meristem for the subsequent formation of floral organs (Wellensiek, 1964). Acquisition of flowering ability is mitotically stable and constitutes a form of cell memory that can be maintained for weeks until optimal light conditions induce bolting (Lang, 1965; Metzger, 1988). Thus, under long-day conditions, bolting, associated with the use of stored sucrose, occurs in vernalized sugar beet and is usually followed by the development of an indeterminate inflorescence. In contrast to sugar beet, wild beets have a facultative requirement for vernalization; both annual and biennial forms have been described (Van Dijk, 2009). The annual habit in beet is under the genetic control of a dominant Mendelian factor termed *B* or ‘*bolting gene*’, which over-rides the need for vernalization by promoting flowering (Abegg, 1936; Boudry *et al.*, 1994; El-Mezawy *et al.*, 2002). Annual beets (*BB* or *Bb*) are non-responsive to vernalization, while biennial beets (*bb*) are responsive to vernalization. Recently, two additional loci affecting bolting, named *B2* and *B4*, were identified. The *B2* locus is unlinked to *B* and appears to act epistatically to *B*, while the *B4* locus is linked to the *B* locus and promotes annual bolting independently of *B* (Büttner *et al.*, 2010; Abou-Elwafa *et al.*, 2012).

Depending on the climate, sugar beet can be grown as a spring crop (temperate climates) or as a winter crop (warmer climates) sown in autumn (Mutasa-Göttgens *et al.*, 2012). The development of winter crop varieties in cooler temperate regions could result in at least a 26% increase in sugar yield (Jaggard and Werker, 1999). However, cool temperatures during the growing season may induce premature vernalization and subsequent early bolting in some genotypes, and reduce sugar production. There is a 12% loss in root sugar yield for one premature bolting plant per square metre in the field (Longden, 1989). Bolting tolerance is a quantitative trait reflecting the bolting response of biennial genotypes after a cold exposure. Several parameters are used to measure bolting tolerance, including bolting index (BI), corresponding to the percentage of bolting plants, and bolting delay (BD), which is the average number of days required for a visible initiation of bolting (Trap-Gentil *et al.*, 2011). At present, the selection of late bolting cultivars is based on phenotypic rather than genotypic criteria, and mechanisms controlling the variation in bolting tolerance between beet genotypes are still unclear.

The genetic control of flowering was first elucidated in the annual model plant *Arabidopsis thaliana* (for a review, see Andrés and Coupland, 2012). In this plant, vernalization involves the epigenetic silencing of the *FLOWERING LOCUS C* (*FLC*) gene. This MADS-box transcription factor acts as a floral repressor inhibiting the expression of genes required to switch the meristem to a floral fate, such as the *FLOWERING LOCUS T* (*FT*) gene which encodes the mobile floral-inductive signal (Michaels and Amasino, 1999; Abe *et al.*, 2005; Searle *et al.*, 2006). The epigenetic silencing of *FLC* is stable and can be transmitted to daughter cells by mitosis, but its expression is restored in each generation (Michaels and Amasino, 2001; Henderson and Jacobsen, 2007). In the last few years, several vernalization genes were identified in sugar beet by homology with counterparts of *Arabidopsis*. Both partial conservation and divergence in the genetic basis of bolting and flowering were found between these two plants (Reeves *et al.*, 2007; Pin *et al.*, 2010, 2012). Indeed, a reverse genetics approach allowed the identification of a *FLOWERING LOCUS C*-like gene (*BvFL1*). The expression of *BvFL1* in sugar beet was shown to be down-regulated during vernalization, but, unlike *AtFLC*, this repression is not maintained after vernalization (Reeves *et al.*, 2007). In addition, two paralogues of the *Arabidopsis FT* gene, named *BvFT1* and *BvFT2*, were isolated and shown to have evolved antagonistic functions (Pin *et al.*, 2010). In biennial sugar beet, *BvFT1* acts as a floral repressor and is gradually down-regulated during vernalization, enabling the induction of *BvFT2*, which is the functional *FT* orthologue and promotes flowering. Recently, the bolting locus *B* was map-based cloned and sequenced, revealing multiple rearrangements between the annual and the biennial alleles and the presence of several linked genes (Pin *et al.*, 2012). One is a pseudo-response regulator gene called *BOLTING TIME CONTROL 1* (*BvBTC1*) acting as an upstream regulator of *BvFT1* and *BvFT2* and being up-regulated by long days in annuals. In biennials, the rare recessive allele *Bvbtc1* retained a role as a promoter of bolting, but imparts reduced sensitivity to photoperiod which is restored by vernalization. In the same way, the *B2* locus was map-based cloned and sequenced, revealing a zinc finger transcription factor gene termed *BvBBX19* that is diurnally regulated and acts like *BvBTC1* upstream of *BvFT1* and *BvFT2* (Dally *et al.*, 2014). Taken together, these data illustrate how evolutionary plasticity at a key regulatory point can enable new life strategies.

Experimental observations, such as post-vernalization bolting of some *Bvbtc1* and *BvFT2* gene knockdown (RNAi) plants, indicate that additional mechanisms are involved in initiation of bolting in sugar beet (Pin *et al.*, 2010, 2012). In addition, the mechanisms controlling the expression of these sugar beet orthologues are still poorly known. As in *Arabidopsis* (for reviews, see Song *et al.*, 2012, 2013; Baulcombe and Dean, 2014), an epigenetic control of vernalization has been shown in sugar beet (Trap-Gentil *et al.*, 2011). Specifically, the time course and amplitude of DNA methylation variations in the shoot apical meristem have been shown to correspond to critical points for the induction of sugar beet bolting and represent an epigenetic component of the genotypic bolting tolerance. Genotypes with different bolting tolerance levels could be distinguished by their global DNA methylation levels in the shoot apical meristem, with sensitive genotypes being hypermethylated compared with resistant genotypes. Recently, a few functional targets of DNA methylation during vernalization such as *RNA METHYLCYTOSINE TRANSFERASES* (*BvRNMT*) were identified in the shoot apical meristem of sugar beet genotypes with distinct bolting tolerance (Hébrard *et al.*, 2013). RNA methylation was shown to vary during cold exposure and between genotypes, to affect *BvFL1* mRNA, and to delay bolting in an *Atrnmt Arabidopsis* mutant.

Here, high-throughput gene expression/methylation analysis of shoot apical meristems from six sugar beet genotypes that were submitted to 9 weeks of vernalization treatment is reported. A total of 169 differentially expressed genes (DEGs) and 111 differentially methylated regions (DMRs) were identified between bolting-resistant and bolting-sensitive genotypes. Two DEGs and 27 DMRs (all with a higher relative methylation level in bolting-resistant genotypes) mapping to the mitochondrial genome were also found. In addition, 14 sequences were found to be both differentially methylated and differentially expressed. It was shown that these DEGs, DMRs, and joint DEG–DMR sequences (DEMSs) belong to an integrated gene network involved in environment perception, phytohormone signalling, and flowering induction. Altogether, the data suggest that a genotype-dependent remodelling of DNA methylation and expression of an integrative gene network participate in bolting tolerance. These results also up open perspectives for breeding and provide the first candidate molecular markers for bolting tolerance in sugar beet.

## Materials and methods

### Plant material and growth conditions

Six biennial sugar beet genotypes (*Beta vulgaris altissima*, provided by SES-VanderHave, Tienen, Belgium) were germinated and grown for 8 weeks at 22 °C under a 16h photoperiod (700 μM m^–2^ s^–1^). Vernalization at 4 °C was then applied for 0, 3, 9, or 18 weeks, as described by Trap-Gentil *et al.* (2011). In this study, the genotypes vernalized for 9 weeks were used for only molecular biology analyses.

### DNA preparation, Cot fractionation, and 454 sequencing

Nuclear DNA was extracted from 500g of sugar beet leaves from the R1 genotype according to Peterson *et al.* (1997). A Misonix Sonicator 3000 (Farmingdale, NY, USA) was used to shear sugar beet nuclear DNA into fragments with a mean length of 500bp. The DNA fragments were dissolved in 0.03M sodium phosphate buffer (SPB) containing 1mM EDTA and loaded onto a hydroxyapatite (HAP) column equilibrated with 0.03M SPB. SPB was prepared as detailed in Liu *et al.* (2011). The absorbance (260nm) of the column eluant was monitored using an Agilent 8530 spectrophotometer (Massy, France). The HAP column was washed with 10 column volumes of 0.03M SPB and 10 column volumes of 0.12M SPB. These washes remove carbohydrate contaminants and secondary compounds that may affect DNA reassociation. The DNA was eluted from the column with the addition of 0.5M SPB. The HAP-purified DNA was concentrated using a Centriplus YM-30 column (EMD Millipore Corporation, Billerica, MA, USA) and sealed in a glass microcapillary tube.

Cot filtration was used to fractionate the HAP-purified sugar beet DNA fragments into single/low-copy and repetitive DNA components. As detailed in Peterson (2005), the genome size of a eukaryote can be used to estimate the Cot1/2 value of its single-copy component using the following formula:

$$y=\left(Cot1/2_{COli}\times G_{org}\right)÷G_{coli}$$ (1)

where *y* is the estimated Cot1/2 of single-copy DNA for the organism of interest (i.e. the Cot value at which half the DNA in the single-copy component has reassociated), G_coli_ is the genome size in base pairs of *Escherichia coli*, G_org_ is the 1C DNA content of the organism of interest in base pairs, and Cot1/2_coli_ is the Cot1/2 of *E. coli* DNA (Peterson, 2005). Inserting *E. coli*’s known genome size (4.639 Mbp; Blattner *et al.* 1997), its Cot1/2 value (4.545455M s^–1^; Zimmerman and Goldberg, 1977), and the 1C DNA content of sugar beet (1.223 Gbp; Bennett, 1972) into Equation 1 and solving for *y* yields an estimated Cot1/2 value for sugar beet single-copy DNA of 1200M s^–1^. Based on the two Cot decade principle (Peterson, 2005), 80% of the single-copy component of the sugar beet genome is expected to reassociate between 0.1*y* and 10*y* (i.e. Cot 120 and Cot 12 000M s^–1^). The concentrated sheared DNA sample in 0.5M SPB (sealed in a glass microcapillary tube as described above) was heated at 95 °C for 10min and allowed to renature to Cot 940M s^–1^. The slowest reassociating component of a Cot curve represents the reassociation of single-copy DNA (Supplementary Fig. S1 available at *JXB* online). It was chosen to reassociate the sample to the fairly stringent value of Cot 940M s^–1^ to ensure that most sequences recovered as unreassociated DNA were truly single copy in nature. The partially reassociated sample was fractionated into single-stranded and double-stranded DNA fractions by HAP chromatography as described by Peterson *et al.* (2002). Those fragments remaining single-stranded at Cot >940M s^–1^ were eluted in 0.5M SPB, concentrated using a Millipore YM-30 column, and transferred into 10mM aqueous TRIS (pH 8.0). Because recovery of Cot >940M s^–1^ was relatively low (i.e. 1–2 μg), the Cot >940 single-stranded DNA was amplified (and concomitantly made double-stranded) using the Qiagen Repli-g Midi Kit (Qiagen, Courtaboeuf, France). The Repli-g amplification resulted in a yield of 185 μg of double-stranded DNA with a concentration of 1840 μg ml^–1^. The *A*_260_/*A*_280_ ratio for the Repli-g amplified DNA was 1.83. Gel electrophoresis indicates that the Repli-g amplification worked well, although it appears to have preferentially amplified the longer fragments in the reactions (> 2kb; data not shown).

The Cot **>**940 DNA was processed for 454 sequencing at GATC-Biotech (Koblenz, Germany) using the GS-FLX technology (Roche, Boulogne Billancourt, France) following established manufacturer’s protocols (Margulies *et al.*, 2005). Half an FLX run was performed. Sequence assembly was achieved using the Roche Diagnostic GS de novo Newbler Assembler, and repeated sequences were eliminated using Repeat Masker software (http://www.repeatmasker.org/).

## Microarray probe design

eArray software (https://earray.chem.agilent.com/earray/; Agilent technologies, Massy, France) was used to design transcriptomics and CH3 microarray probes corresponding to 60-mer oligomers based on the 42 004 predicted open reading frames (ORFs) and 6231 contigs, respectively, and on public sequence information from a large collection of sugar beet expressed sequence tags (ESTs; ~30 000) and the mitochondrial genome from the NCBI (http://www.ncbi.nlm.nih.gov/nucest/?term=beta%20vulgaris). Sugar beet genes known to be involved in the floral transition were added, such as *FLC* (Reeves *et al.*, 2007), *FT* (Pin *et al.*, 2010), or *CONSTANS-LIKE* (Chia *et al.*, 2008). Previous studies on sugar beet revealed 39 vernalization markers (Hébrard *et al.*, 2013) and 21 morphogenesis markers (Maury *et al.*, 2012) which were also included.

At least one transcriptomic probe was designed for each sugar beet EST, ORF, or gene. A second probe was designed when probes could not differentiate between sequences. A total of 53 752 probes were generated with this design, and 1227 probes allowed the differentiation between similar transcripts. Moreover, internal Agilent control probes and reproducibility controls, corresponding to 50 probes that were duplicated 10 times, were also used. For hybridization, 8×60K custom microarray slides (Agilent Technologies) were used.

The design of CH3 microarray probes was different depending on defined groups. The probe coverage was maximal, but the space between probes varied for each group. In the first and second groups, corresponding to public sugar beet EST data and the mitochondrial genome, respectively, probes were designed every 100bp. The contigs obtained by sequencing of Cot-filtered DNA, vernalization, and morphogenesis markers were covered by 50bp spaced probes. Candidate genes and ORF probes were designed every 30bp. Moreover, internal Agilent control probes and reproducibility controls, corresponding to 50 probes that were duplicated 10 times, were also used. Finally, 227 242 probes were designed and 1×244K custom microarray slides were used (Agilent Technologies) for hybridization.

### RNA preparation and microarray hybridization

Total RNAs were independently isolated from shoot apical meristems after 9 weeks of vernalizing treatment in three bolting-sensitive (S1, S2, and S3) and three bolting-resistant (R1, R2, and R3) sugar beet genotypes using a Nucleospin^®^ RNA Plant kit (Macherey-Nagel, Hoerdt, France). One shoot apical meristem per genotype was used. To confirm the absence of genomic DNA in all the RNA preparations, a control PCR was performed on RNA. The DNA contamination and RNA integrity with RNA integrity number (RIN) measurement (RIN threshold of 8) were estimated by the use of a bioanalyzer 2100 (Agilent Technologies).

Sugar beet total RNA was used to generate fluorescent cRNA (complementary RNA), according to Agilent Technologies’ recommendations. Briefly, to be used as a positive control for labelling and data analysis, RNA spike mix, which is a mix of 10 polyadenylated RNAs synthesized *in vitro* from the *E1A* adenovirus gene, was obtained with the One-Color RNA Spike-In Kit (Agilent Technologies). For each sample, 80ng of total RNAs were reverse transcribed. Then, cRNA was synthesized by T7 RNA polymerase, and cyanine 3 (Cy3)-labelled CTP was incorporated using an Input Quick Amp Labeling Kit, One Color (Agilent Technologies). Fluorescent cRNA was purified with the RNeasy Mini Kit (Qiagen). Quality control of synthesized fluorescent cRNA was based on cRNA and Cy3 concentration measurement as well as cRNA fragment size. For each sample, three quality thresholds were chosen: a yield of 825ng of cRNA, a specific activity of 6 pmol_Cy3_ g_cRNA_^–1^, and a 200–2000 nucleotides fragment size range.

Imaxio (Clermont-Ferrand, France) conducted RNA hybridization following Agilent Technologies’ instructions. Briefly, the labelled cRNAs were broken up in Fragmentation mix (Agilent Technologies) for 30min at 60 °C. A 2× GEx Hybridization Buffer HI-RPM (Agilent Technologies) was then added and cRNAs were loaded onto the arrays. The arrays were incubated at 65 °C for 17h in Agilent hybridization chambers and then washed according to the Agilent protocol at room temperature.

### DNA preparation and microarray hybridization

Shoot apical meristems from three bolting-sensitive (S1, S2, and S3) and three bolting-resistant (R1, R2, and R3) sugar beet genotypes exposed to 9 weeks of vernalization were used for DNA isolation. For each genotype, shoot apical meristems from three individuals were pooled, ground into a fine powder in liquid nitrogen, and used for DNA extraction. DNA was extracted using Plant DNAzol Reagent (InVitrogen, Cergy-Pontoise, France) according to the manufacturer’s recommendations. To avoid RNA contamination, 100 μg of RNase A ml^–1^ Plant DNAzol was added at the beginning of the isolation procedure. DNA was precipitated at –20 °C for at least 30min using 96% ethanol (2.5 vols) and 3M Na-acetate (pH 5.2, 0.1vol.). DNA was then washed using 70% ethanol, vacuum dried, and dissolved in ultrapure water. Concentrations and purity of DNA samples were estimated using a NanoDrop 1000 spectrophotometer (Thermo Scientific, Wilmington, DE, USA).

Sugar beet DNA fragments ranging from 0.2kb to 0.8kb were obtained by sonication with a VC 505 Vibra-Cell sonicator (Fisher Scientific, Illkirch, France). DNA was isolated by immunoprecipitation according to Weng *et al.* (2009). One immunoprecipitation per DNA sample was realized. Briefly, 11 μg of sonicated DNA were diluted in 250 μl of 1× IP buffer composed of 10mM Na_3_PO_4_ (pH 7.0), 140mM NaCl, 0.05% (v/v) Triton X-100, and denaturated by boiling for 10min. Then DNA was cooled on ice for 10min and 4 μg of DNA from each sample was kept at –20 °C to be used as the input control for microarray analysis. The remaining 7 μg of DNA of each sample was mixed with 10 μg of mouse monoclonal anti-5mC antibodies (Eurogentec, Angers, France) and incubated overnight on a rotator in a 4 °C cold room. The DNA–antibodies mixture was combined with Dynabeads Protein G (InVitrogen) during 2h at 4 °C. Beads were washed four times with 1× IP buffer using a magnetic rack (Invitrogen). Antibodies were digested overnight on a rotator with 100 μg of proteinase K (Sigma-Aldrich, Saint-Quentin Fallavier, France) in 250 μl of a digestion buffer composed of 50mM TRIS (pH 8.0), 10mM EDTA, and 0.5% (w/v) SDS at 50 °C to elute methylated DNA. Isolated DNA was obtained with one phenol/chloroform/isoamyl alcohol (25:24:1) (v/v/v) and two chloroform/isoamyl alcohol (24:1) (v/v) extractions in phase lock gel heavy tubes (Eppendorf, Le Pecq, France) followed by ethanol precipitation. A NanoDrop 1000 spectrophotometer (Thermo Scientific) was used to estimate concentrations of DNA pellets that were solubilized in ultrapure water.

For each sample, labelled DNA was generated according to the recommendations of Agilent Technologies. Briefly, labelling of input control and immunoprecipitated methylated DNA was performed using a Genomic DNA Enzymatic Labeling Kit (Agilent Technologies). Random primers and the exo-Klenow fragment were used to label input control and immunoprecipitated methylated DNA differentially with Cy3-labelled dUTP and Cy5-labelled dUTP, respectively. Labelled DNA was purified using 30kDa Amicon columns (Millipore, Molsheim, France). Quality control of synthesized labelled DNA was based on DNA and Cy5/Cy3 concentration measurement. For each sample, three quality minimum thresholds were chosen: a yield of 2.5 μg of labelled DNA, a specific activity of 7–20 pmol_Cy5_ g_DNA_^–1^, and 18–25 pmol_Cy3_ g_DNA_^–1^.

Imaxio (Clermont-Ferrand, France) conducted DNA hybridization following Agilent Technologies’ instructions. Briefly, Cy5- and Cy3-labelled DNAs were pooled in ddH_2_O, and Hybridization mix (Agilent Technologies) was added before incubation at 95 °C for 3min and then at 37 °C for 30min. Samples were loaded onto the arrays, and hybridization was performed at 67 °C for 40h at 20rpm in Agilent hybridization chambers. Arrays were then washed according to the protocol at room temperature.

### Data collection, normalization, and cluster analysis

The single nucleotide polymorphism (SNP) collection was obtained in a previous study (Adetunji *et al.*, 2014) by genotyping elite sugar beet breeding lines (from SESVanderHave) and wild beet accessions.

A G2505C scanner (Agilent Technologies) was used to scan microarray slides, and extraction of raw signal data was carried out using Feature Extraction 10.7 software. The reproducibility and reliability of each single microarray were evaluated using Quality Control report data (Feature Extraction, Agilent Technologies).

One-colour transcriptomics data were normalized per chip to the 75th percentile and per probe to medians and were transformed to log values. Feature Extraction flag criteria allowed data filtering: non-outlier population, not saturated; uniform and significant probe signal, probe signal above background. A probe signal was considered as ‘well above background’ if gBGSubSignal was >2.6 gBG_SD. A probe needed to pass all flag criteria for all replicates of at least one experimental condition (bolting-resistant or bolting-sensitive) to pass the filtering. A *t*-test was performed with MeV software (Saeed *et al.*, 2003) to determine if genes were differentially expressed between experimental conditions. Statistical tests were considered significant at *P<0.05. Benjamini–Hochberg’s correction was used for false-positive discovery. The second filtering criterion which was used is a 2-fold change in transcript levels among conditions. Average linkage hierarchical clustering analysis was applied using Pearson correlation distance.

For the CH3 microarray, data normalization was performed by Feature Extraction software and used to correct cyanine bias with the linear method: the mean signal of probes selected for quality criteria (i.e. signal above background, non-outlier, and whose ranks are similar between the two dyes) allowed the normalization of all probes values. The Cy5/Cy3 log ratio was then calculated for each probe. Data filtering was performed using Feature Extraction flag criteria: non-outlier population, not saturated; uniform and significant Cy3 and Cy5 probe signal, Cy3 and Cy5 probe signal above background. A probe signal was considered as ‘well above background’ if g/rBGSubSignal was >2.6g/rBG_SD. A probe needed to pass all flag criteria for all replicates of at least one experimental condition (bolting-resistant or bolting-sensitive) to pass the filtering. A *t*-test was performed with MeV software (Saeed *et al.*, 2003) to determine if genes were differentially methylated between experimental conditions. Statistical tests were considered significant at *P<0.05. A probe signal fold change of two between the two experimental conditions was also chosen as a threshold.

### Bioinformatic analyses

Reads were mapped on the sugar beet genome (Dohm *et al.*, 2014; The Beta Vulgaris Resource: bvseq.molgen.mpg.de) using CLC Genomics Workbench software (Qiagen). Contigs and ORFs were compared with the sugar beet genome using BLASTN. Differentially expressed and differentially methylated genes were annotated (i) by TBLATSX analyses using the *Arabidopsis* database from the National Center for Biotechnology Information (http://blast.ncbi.nlm.nih.gov/Blast.cgi); and (ii) by BLASTX analyses using The Beta Vulgaris Resource (bvseq.molgen.mpg.de). The Gene Ontology (GO) class enrichment was produced with Classification SuperViewer Tool w/ Bootstrap (http://bar.utoronto.ca/ntools/cgi-bin/ntools_classification_superviewer.cgi). These analyses were confirmed using Blast2Go (http://www.blast2go.com/b2ghome; Götz *et al.*, 2008).

### Quantitative PCR

Approximately 500ng of total RNA were reverse transcribed using SuperScript™ III First-Strand Synthesis SuperMix for qRT-PCR kit (Invitrogen). Primers were designed with QuantPrime software (http://www.quantprime.de/) and are listed in Supplementary Table S1 at *JXB* online. Constitutively expressed genes encoding, respectively, a *UBIQUITIN* and an *ATP SYNTHASE BETA SUBUNIT* (accession nos *BI544002* and *BQ490219*) were used as internal standards. To confirm the absence of genomic DNA in all the total RNA preparations, a control without reverse transcriptase during the cDNA synthesis was performed. PCRs were prepared using an automated system epMotion 5070 (Eppendorf). Amplification and analysis were performed using a Platinium SYBR Green qPCR Supermix-UDG kit (Eurogentec) on a Mastercycler Realplex (v1.6000; Eppendorf). Two biological and two technical replicates at least were performed for each gene and for six genotypes (three resistant and three sensitive to bolting). Quantitative PCR and microarrays results were compared calculating the Pearson correlation coefficient between qPCR amplification ratios and fold changes.

### Bisulphite sequencing

To determine the cytosine methylation status in CG, CHG, or asymmetric CHH (H could be A, T, or C) contexts, bisulphite treatment (Epitect bisulfite kit, Qiagen), which results in the conversion of unmethylated cytosines to uracils but does not affect methylated cytosines, was applied on genomic DNA (500ng) according to the manufacturer’s recommendations. Controls for bisulphite conversion efficiency and amplification as well as primer design, PCR, and sequencing procedures have been previously detailed (Trap-Gentil *et al.*, 2011; Hébrard *et al.*, 2013). Primers used for bisulphite sequencing are listed in Supplementary Table S1 at *JXB* online. Duplicates were performed for each sequence and each genotype.

## Results

### Isolation of sugar beet single-copy sequences by Cot filtration and next-generation sequencing (Cot-SEQ)

In order to obtain more gene sequence data for sugar beet, which has a 1223 Mbp genome (Bennett, 1972) containing >63% repetitive DNA (Flavell *et al.*, 1974), Cot filtration was used to isolate single/low-copy sequences which were subsequently sequenced using 454 technology (Supplementary Fig. S2 at *JXB* online). An average read length of 353bp was obtained for the 466 386 reads, corresponding to 117 949 495 nucleotides (Supplementary Fig. S3, Supplementary Table S2). The 466 386 reads were assembled into 6231 contigs, including 2211 large contigs (>500 bases, 832 bases average size, 14 860 bases largest size), and covered 2 953 454 bases (Supplementary Table S2). Large contigs have been deposited at DDBJ/EMBL/GenBank under the accession no. JMBQ00000000. About 78% of the total reads remained as singletons. Bioinformatic analyses revealed that single-copy sequences represented 98.6% of the covered bases while repeated sequences covered only 1.4%, mostly with small RNAs and simple repeats (Supplementary Table S3). Only 1.0% of the contigs (Supplementary Table S4) were mapped on the recent version of the reference sugar beet genome. Analysis of contigs identified thousands of potential ORFs that were blasted against public databases. These ORFs revealed very low sequence redundancy (1.5%), and 3.9% showed significant homology to previously annotated genes (Supplementary Tables S3, S5). GO annotations indicate that those sequences with homology to known genes represent a wide range of biological processes (Supplementary Fig. S4A) and molecular functions (Supplementary Fig.S4B). ORFs were then mapped on the sugar beet genome when it was released (Dohm *et al.*, 2014), and 0.52% of ORFs (i.e. 218) were mapped on the sugar beet genome (Supplementary Table S6). Among them, 184 ORFs showed sequence homology to genes, but only 84 distinct genes were identified. Using a Cot-SEQ strategy, information on single/low-copy sequences that were previously undescribed was generated and thus additional information was provided that was leveraged in designing microarrays for sugar beet.

### Bolting tolerance and genetic clustering of sugar beet genotypes

**Fig. 1.**
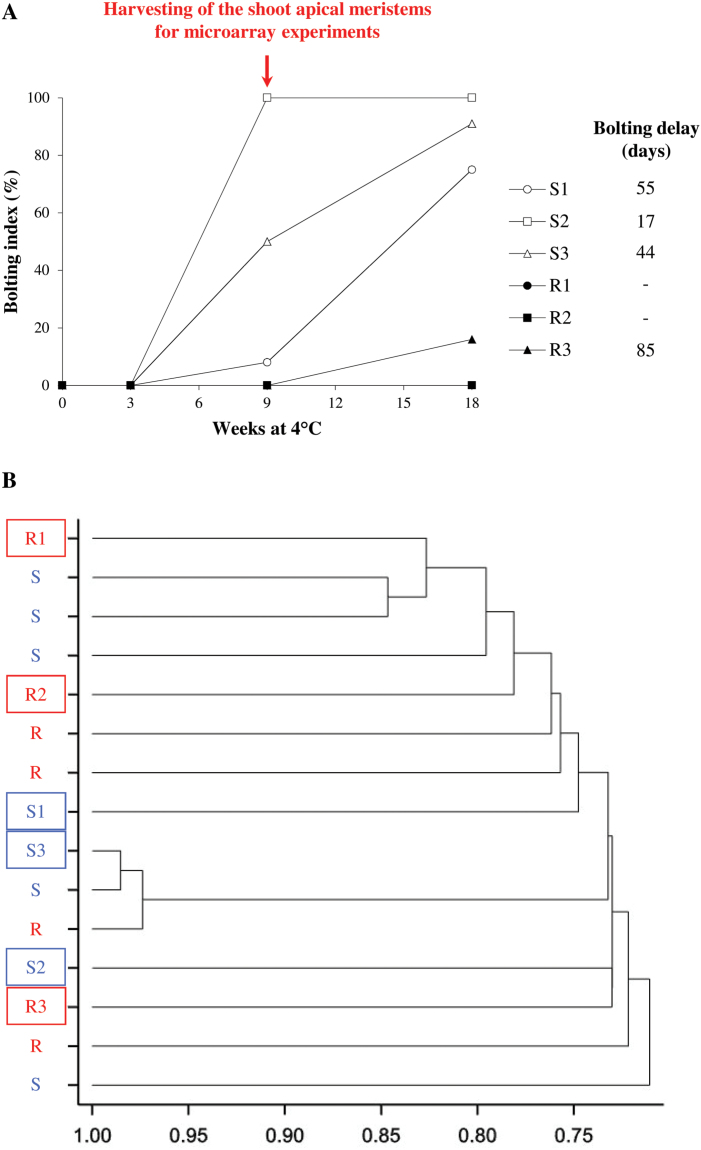
Characterization of the six sugar beet genotypes during vernalization. (A) Bolting index of genotypes after 0, 3, 9, or 18 weeks of vernalization treatment (4 °C). S1, S2, and S3 (open circles, squares, and triangles) were considered as bolting sensitive, while R1, R2, and R3 (filled circles, squares, and triangles) were considered as bolting resistant. On the right is indicated the bolting delay after 18 weeks of vernalization for each genotype. (B) Genetic clustering based on 708 single nucleotide polymorphisms for the three bolting-resistant (R1–R3) genotypes, the three bolting-sensitive (S1–S3) genotypes, and nine other genotypes with distinct bolting tolerance levels after 9 weeks of vernalization treatment.

Six sugar beet genotypes were kept at 4 °C for up to 18 weeks and then characterized for bolting using the percentage of bolting plants (the bolting index, BI) after 0, 3, 9, or 18 weeks of cold and the average number of days required for bolting initiation (the bolting delay, BD; Fig. 1A). The BI increased with the duration of cold, depending on the genotypes. Genotypes S1, S2, and S3 started to bolt after 9 weeks at 4 °C and reached 75–100% of the BI at the end of the treatment, with BDs ranging from 17 d to 55 d. Conversely, genotypes R1, R2, and R3 did not bolt, or showed weak bolting responses during the treatment (R3 with a 15% BI and BD of 85 d after 18 weeks of cold exposure). Therefore, S1–S3 genotypes were considered as bolting sensitive compared with R1–R3 which were classified as bolting resistant. The shoot apical meristems were collected after 9 weeks of cold exposure for further microarray analyses. Indeed, this cold duration was sufficient to induce bolting in the three bolting-sensitive genotypes when transferred to optimal temperature and photoperiod conditions, but not in the three resistant genotypes.

A hierarchical genetic clustering (Fig. 1B) based on 708 SNPs (explaining ~46% of the variance) showed that the six genotypes were classified independently from their bolting characteristics. These SNPs were obtained from a previous study by genotyping elite sugar beet breeding lines (from SESVanderHave) and wild beet accessions (Adetunji *et al.*, 2014).

### Trancriptomic microarray analysis of vernalized sugar beet genotypes with distinct bolting tolerance

To explore transcriptomic variations between sugar beet genotypes with distinct bolting tolerance levels, a custom long-oligonucleotide (60-mer) microarray (8×56k) was generated using the Agilent SurePrint ink-jet technology and hybridized with total RNA extracted from the shoot apical meristems of each genotype after 9 weeks of cold exposure (Supplementary Fig. S2 at *JXB* online). Transcriptomic data are available in the GEO database (GEO accession no. GSE55951).

**Fig. 2.**
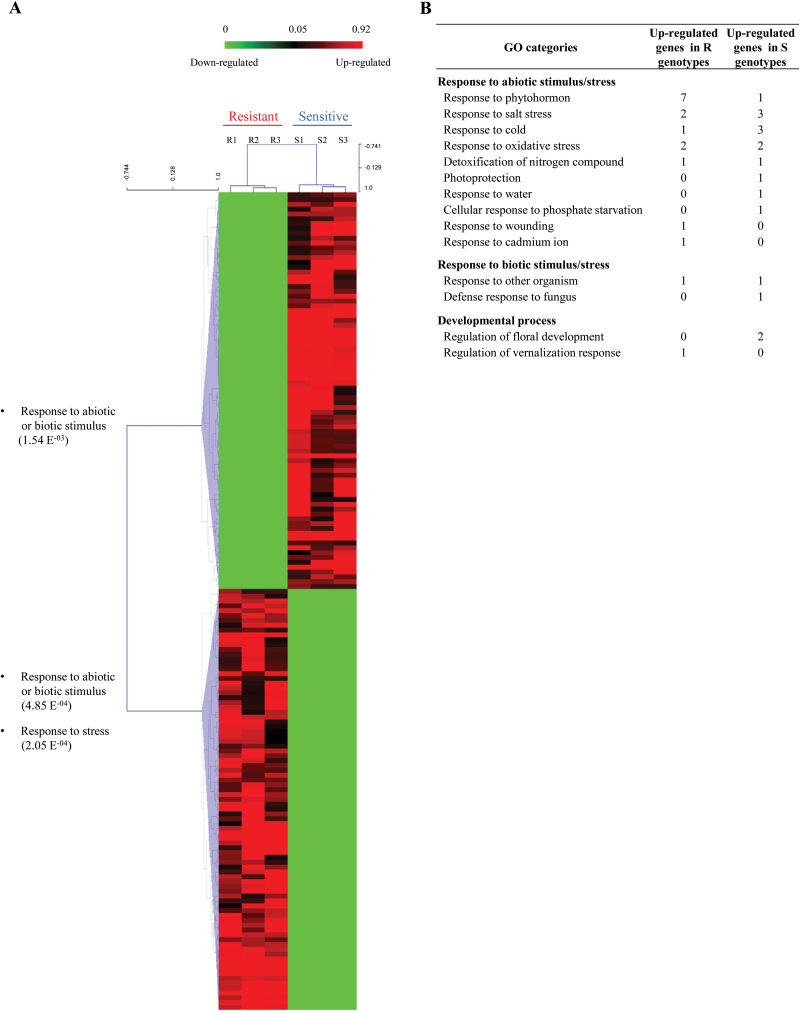
Transcriptomic characterization of the shoot apical meristem in sugar beet genotypes after vernalization. (A) Heatmap representation of the 169 differentially expressed genes (fold change R versus S >2 and *t*-test *P*-value <0.05) between bolting-resistant (R1–R3) and bolting-sensitive (S1–S3) genotypes after 9 weeks of vernalization treatment. On the left are indicated the main biological processes in which sequences could be involved and the corresponding *P*-value. (B) GO categories for genes involved in response to abiotic or biotic stimulus/stress and developmental process. (This figure is available in colour at *JXB* online.)

Of the 53 752 probes (29 094 from public databases and 24 658 from Cot-SEQ; Table 1), 22 448 (41.8%) remained after the filtration and normalization steps (Supplementary Fig. S5 at *JXB* online), mainly from the public databases probes. Using a fold change threshold of two, 1580 genes were found to be differentially expressed between S and R genotypes. According to a *t*-test (*P*<0.05), 169 of these 1580 genes revealed significant differences between genotypes and were considered as ‘differentially expressed genes’ (DEGs; Table 1; Supplementary Table S7). Microarray transcript abundance data were independently validated by quantitative reverse transcription–PCR (qRT–PCR) for a sample of genes (Supplementary Fig. S6), revealing a strong positive correlation between fold changes obtained from microarray and qPCR data (Pearson correlation coefficient *r*=0.986 at *P*<0.005, *n*=6). Figure 2A corresponds to a heatmap showing the 82 sequences with a higher relative expression level in S genotypes and the 87 sequences with a higher relative expression level in R genotypes. Genes with a lower relative expression level exhibited homogeneous expression levels between genotypes of the same group (R or S), while genes with a higher relative expression level exhibited variations among genotypes, allowing a clustering in agreement with their bolting tolerance (Fig. 2A).

**Table 1.** Number of transcriptomic and methylation probes after each step of the microarray design and analysis

	Origin of the sequences	Submitted sequences	Designed probes	Filtered probes	DEGs or DMRs
Transcriptomic	Public database	154 mitochondrial ORFs	132	106	2
		30 023 ORFs from ESTs	28 918	21 043	159
		22 candidate genes	44	26	3
	Cot fraction	42 004 ORFs	24 658	1273	5
	Total	72 203	53 752	22 448	169
Methylation	Public database	1 mitochondrial genome	3688	3211	27
		29 585 ESTs	185 425	14 069	110
		22 candidate genes	1426	109	1
	Cot fraction	6231 contigs	36	2185	0
	Total	35 839	227 242	19 574	138

DEG, differentially expressed gene; DMR, differentially methylated region. The Cot fraction corresponds to single-copy sequences obtained after Cot filtration and 454 sequencing of sugar beet genomic DNA. DEGs and DMRs correspond to sequences showing both significantly different signals (*t*-test analysis with *P*<0.05 and *P*<0.01, respectively) and fold change >2 between resistant and sensitive genotypes.

TBLASTX analysis of the 169 DEGs revealed annotations for 125 of them using the *Arabidopsis* gene database (Supplementary Table S7 at *JXB* online). Based on the TAIR percentage analysis, GO classification of these 125 sequences according to the ‘Biological Process’ component placed the largest proportion of genes in undefined categories such as ‘Other metabolic processes’ (31.9%), ‘Unknown biological process’ (31.9%), ‘Other cellular processes’ (27.9%), but also in ‘Response to abiotic and biotic stimulus’ (24.5%), ‘Response to stress’ (24.5%), and ‘Developmental process’ (12.0%; data not shown). These three last categories corresponded to 34 distinct sequences, including 17 sequences that were up-regulated in R genotypes and 17 in the S genotypes (Fig. 2B; Supplementary Table S7). Among them, eight genes could be regrouped in a ‘Response to phytohormones’ class (seven sequences up-regulated in R and one in S genotypes). Of the four sequences that were grouped together in the ‘Response to cold’ class, three had a higher relative expression level in S and one had a higher relative expression level in R. To validate the experimental approach, sequences directly involved in flowering regulation were studied, and three of them were found to have a higher relative expression level in S [*SHORT VEGETATIVE PHASE* (*BvSVP*) and *AGAMOUS-LIKE 24* (*BvAGL24*)] and one a higher relative expression level in R [*FRUITFULL* (*BvFUL*); Table 2]. Altogether, the bolting tolerance transcriptomic analysis revealed an integrated network of genes that are involved in environmental perception (cold), phytohormone signalling, and flowering induction, and have a distinct relative expression level between R and S genotypes.

**Table 2.** Sugar beet genes involved in the flowering pathway showing significant normalized values (>|0.5|) for their methylation and expression levels

Accession	Gene	Reference	Methylation				Expression	
			Position of probes groups	No. of probes per group	Methylation level in S^*a*^	Methylation level in R^*a*^	Expression level in S^*b*^	Expression level in R^*a*^
HR807147	*RNA METHYLCYTOSINE TRANSFERASE b* (*BvRNMTb*)	Hébrard *et al.* (2013)	0–260	2	1.64	0.58	–	–
BQ592158	*FLOWERING LOCUS VE* (*BvFVE*)	Abou-Elwafa *et al*. (2010)	60–120	1	1.60	0.63	0.58	0.00
HM448911	*FLOWERING LOCUS T2* (*BvFT2*)	Pin *et al.* (2010)	0–240	5	2.25	0.94	–	–
			210–630	9	–2.25	–2.24		
			1080–2070	17	1.42	0.75		
			2820–4260	6	1.31	0.41		
HM448909	*FLOWERING LOCUS T1* (*BvFT1*)	Pin *et al.* (2010)	60–260	3	1.60	1.39	–	–
			1100–1773	8	1.48	0.59		
			2763–4893	6	0.92	0.28		
DQ189214 DQ189215	*FLOWERING LOCUS C (BvFL1*)	Reeves *et al.* (2007)	790–1090	2	1.46	1.00	–0.17	0.30
			5950–6130	5	–2.58	–2.56		
BQ584677	*FRUITFULL* (*BvFUL*)	This study (e-value 6E-83)	–	–	–	–	–0.45	0.55
BQ588867	*SHORT VEGETATIVE PHASE* (*BvSVP*)	This study (e-value 7E-22)	–	–	–	–	0.00	–0.65
EU437782	*CONSTANS* (*BvCOL1*)	Chia *et al.* (2008)	–	–	–	–	0.65	0.36
BQ488144	*AGAMOUS-LIKE 24* (*BvAGL24*)	This study (e-value 4E-30)	–	–	–	–	1.04	–0.75

^*a*^Average of the methylation levels for the indicated number of probes and for the three bolting-sensitive (S) or bolting-resistant (R) genotypes.

^*b*^ Average of expression levels for the three S or R genotypes.

–, The corresponding probes were eliminated after filtration.

### Methylation microarray analysis of vernalized sugar beet genotypes with differing bolting tolerance

To explore epigenetic variation between sugar beet genotypes with distinct bolting tolerance levels, a custom long-oligonucleotide (60-mer) microarray (1×244k) was generated using the Agilent SurePrint ink-jet technology and hybridized with either total or methylated DNA (isolated by methylated DNA immunoprecipitation; see the Materials and methods for details) extracted from the shoot apical meristems of each genotype after 9 weeks of cold exposure (Supplementary Fig. S2 at *JXB* online). Epigenetic data are available in the GEO database (GEO accession no. GSE56415).

**Fig. 3.**
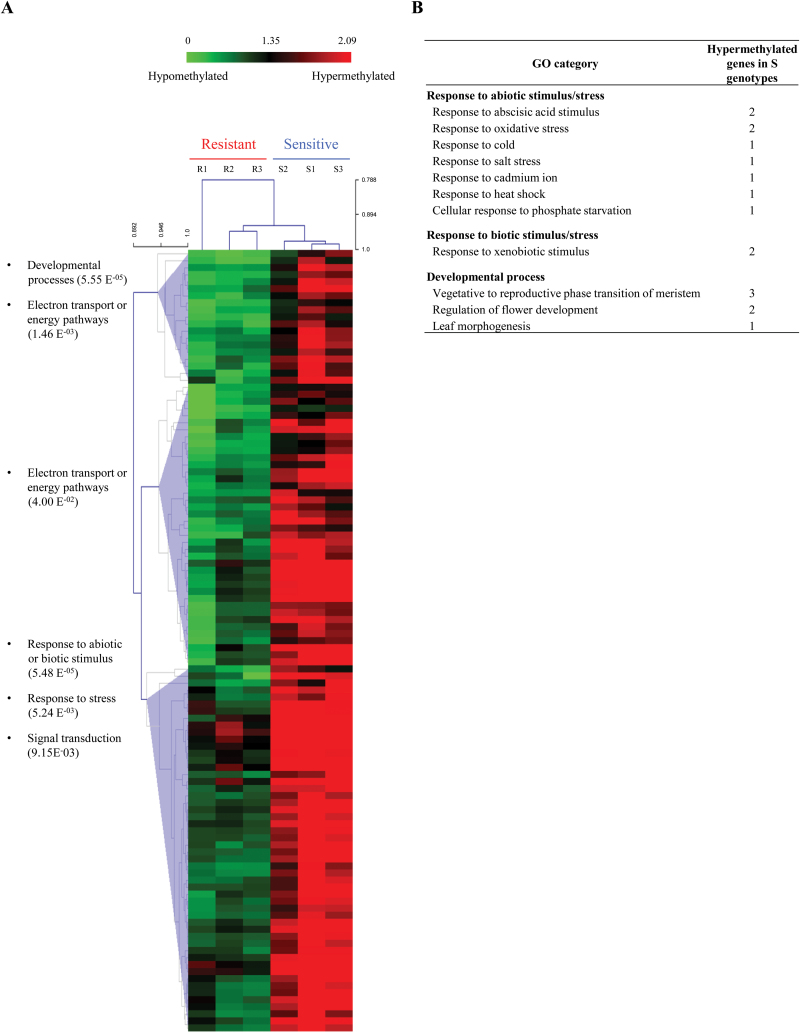
Methylation characterization of the shoot apical meristem in sugar beet genotypes after vernalization. (A) Heatmap representation of the 111 differentially methylated regions (fold change R versus S >2 and *t*-test *P*-value < 0.01) between bolting-resistant (R1–R3) and bolting-sensitive (S1–S3) genotypes after 9 weeks of vernalization treatment. On the left are indicated the main biological processes in which sequences could be involved and the corresponding *P*-value. (B) GO categories for genes involved in response to abiotic or biotic stimulus/stress and developmental process. (This figure is available in colour at *JXB* online.)

Of the 227 242 probes (190 539 from public databases and 36 703 from Cot-SEQ; Table 1), 19 574 (8.6%) remained after the filtration and normalization steps (Supplementary Fig. S7 at *JXB* online), with 11.1% from Cot-SEQ probes. Using a fold change threshold of two, 1526 sequences were found to differ for their methylation levels between S and R genotypes. A *t*-test (*P*<0.05) revealed 707 out of these 1526 sequences that showed significant differences between genotypes, and 111 of them (with *P*<0.01) were considered as ‘differentially methylated regions’ (DMRs; Table 1; Supplementary Table S8). The DNA methylation ratios detected in this microarray experiment were independently validated by bisulphite sequencing (Supplementary Fig. S8). A heatmap is shown in Fig. 3A with the 111 sequences which all had a higher relative methylation level in S genotypes, allowing a clustering in agreement with the bolting tolerance of the six genotypes.

From the 111 DMRs, TBLASTX analysis detected 50 with annotations in the *Arabidopsis* gene database (46 with known function and four uncharacterized; Supplementary Table S8 at *JXB* online). Based on the TAIR percentage analysis, GO classification of these 50 sequences according to the ‘Biological Process’ component placed the largest proportions of genes in undefined categories such as ‘Other metabolic processes’ (18.9%) and ‘Other cellular processes’ (17.6%), but also in ‘Developmental process’ (8.8%), ‘Response to abiotic and biotic stimulus’ (8.1%), and ‘Response to stress’ (7.0%; data not shown). These three last categories corresponded to 17 distinct sequences, all with a higher relative methylation level in S genotypes (Fig. 3B). Among them, nine sequences could be grouped in a ‘Response to abiotic stimulus/stress’ class and six sequences in ‘Developmental process’. To validate the experimental approach, sequences directly involved in flowering regulation were studied, and five of them were found to be significantly hypermethylated in S [such as *FLOWERING LOCUS VE* (*BvFVE*); Table 2]. The methylation data indicated that bolting tolerance is associated with a gene network involved in the response to environment (cold) or in the induction of flowering, which exhibits distinct methylation states between S and R genotypes, these genes having a higher relative methylation level in S genotypes.

### Transcriptomic and methylation microarray analysis of the sugar beet mitochondrial genome

**Fig. 4.**
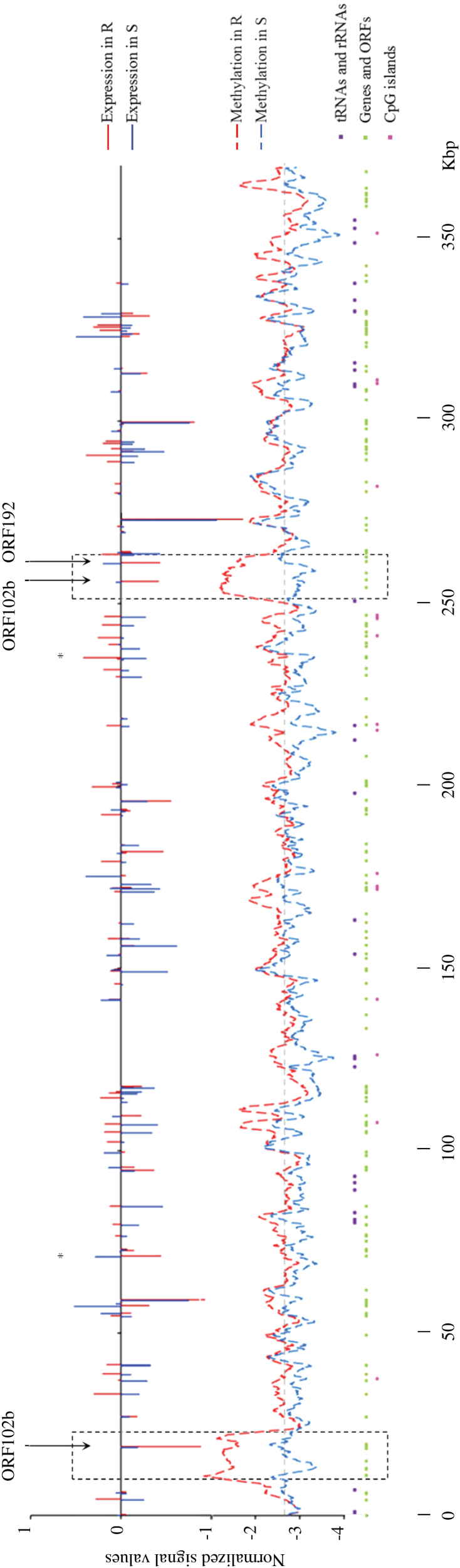
Expression and DNA methylation level analysis in the mitochondrial genome of sugar beet genotypes after vernalization. The three bolting-resistant (R, red lines) and the three bolting-sensitive (S, blue lines) genotypes have the same mitochondrial haplotype (‘O’ type, i.e. normal type mitochondrial genome leading to fertility, in contrast to the cytoplasmic male-sterile type) and were vernalized during 9 weeks at 4 °C. Green squares represent ORFs, purple squares represent rRNAs and tRNAs, and pink squares represent CpG islands. Black frames correspond to differentially methylated region-rich loci. Significantly different values for transcriptomic probes are marked with an asterisk (*P<0.05).

The sugar beet mitochondrial genome (368 kbp; GenBank accession no. BA000009) was used to design oligonucleotides for the custom transcriptomic (132 probes) and methylation (3688 probes) microarrays. After normalization, 106 transcriptomic probes and 3211 methylation probes were kept (Table 1) and mapped on the mitochondrial genomic map for R and S groups (Fig. 4). After filtration (*t*-test at *P*<0.05), only two DEGs were identified: *ORF104a* and *CYTOCHROME OXIDASE C subunit 2* (*COX2*). DNA methylation probes showed a succession of peaks all along the genome in both S and R genotypes, but R genotypes had a significantly higher relative methylation level compared with the S lines (at *P*<0.001). The non-coding regions also had a significantly higher relative methylation level compared with the coding regions (at *P*<0.01). After filtration (*t*-test at *P*<0.05), 27 DMRs (Table 1) were found, with 18 of them mapping on two main loci containing three ORFs (*ORF152*, *ORF102b*, and *ORF192*) that exhibited lower normalized expression values in R genotypes than in S genotypes. These data demonstrated that the mitochondrial genome is also affected by transcriptomic and epigenetic remodelling between sugar beet genotypes.

### Candidate sequences related to the genotypic bolting tolerance

**Fig. 5.**
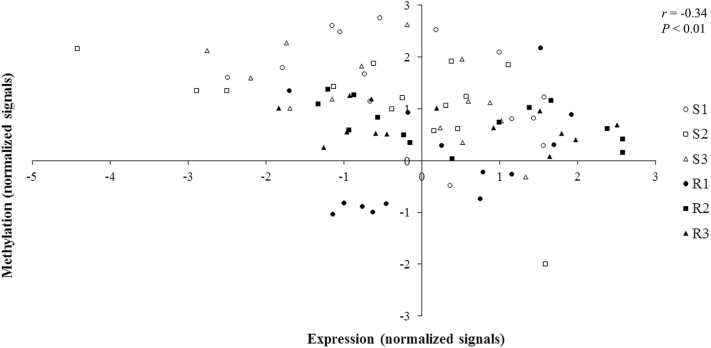
Relationship between methylation and expression in sugar beet genotypes after 9 weeks of vernalization treatment for the 14 sequences that are both differentially methylated (fold change R versus S >2) and differentially expressed (fold change R versus S >2 and *P*<0.05) between resistant and sensitive genotypes. The *x*- and *y*-axis correspond to expression and methylation normalized signals, respectively. Open circles, squares, and triangles represent the three bolting-sensitive genotypes, filled circles, squares, and triangles correspond to the bolting-resistant genotypes.

**Fig. 6.**
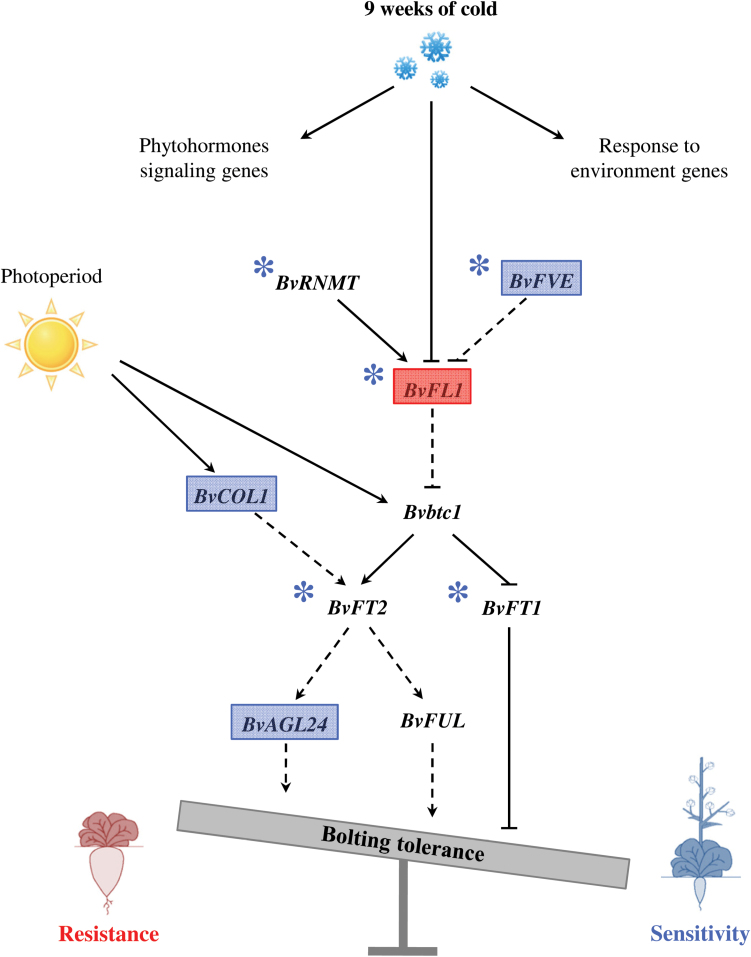
Bolting/flowering model in sugar beet. *BvAGL24*, *AGAMOUS-LIKE 24*; *Bvbtc1*, *bolting time control 1*; *BvCOL1*, *CONSTANS-LIKE 1*; *BvFL1*, *FLOWERING LOCUS C*; *BvFT1* andt *BvFT2*, *FLOWERING LOCUS T 1* and *2*; *BvFVE*, *FLOWERING LOCUS VE*; *BvFUL*, *FRUITFUL*; *BvRNMT*, *RNA METHYLTRANSFERASE*. Arrows and a dotted line indicated a hypothetical control based on the *Arabidopsis* flowering pathway. Genes coloured in blue are floral inducers overexpressed in bolting-sensitive genotypes. *BvFL1*, in red, is a floral inhibitor overexpressed in bolting-resistant genotypes. Asterisks (*) indicate hypermethylated genes in bolting-sensitive genotypes.

In order to identify new candidate genes for bolting tolerance, the 14 sequences found to be both differentially methylated and differentially expressed between R and S genotypes were selected (Table 3). TBLASTX analyses proposed significant annotations for nine of them: four are involved in biosynthesis of various compounds such as suberin and cutin polymers (FG345845, a *HXXXD-TYPE ACYL-TRANSFERASE-LIKE PROTEIN*), triterpenoids (FG343208, a *BETA-AMYRIN SYNTHASE*), carotenoids (BQ582907, a *CAROTENOID EPSILON-RING HYDROXYLASE LUT1/CYP97C1*), or ethylene (FG344953, a *1-AMINOCYCLOPROPANE-1-CARBOXYLATE OXIDASE-LIKE PROTEIN*; Table 3); BQ586261, BQ489455, and BQ585496 encode a *GERMIN-LIKE PROTEIN 5* (*GLP5/PDGLP1*), an *ATPASE E1-E2*, and an *SPX DOMAIN CONTAINING PROTEIN 4* (*SPX4*), respectively, and are involved in early development, endoplasmic reticulum to Golgi vesicle-mediated transport, and response to phosphate starvation, respectively. Interestingly, one sequence is involved in response to environmental stress including cold (FG345694, a *CYSTATIN 6 CYSB*), and another one in the transcriptional regulation of specific signalling pathways such as flowering time (BQ582428, *RNA POLYMERASE II TRANSCRIPTION MEDIATOR*; Table 3). A correlation between methylation and expression values has been detected for these 14 sequences (Pearson *r*= –0.34 and Spearman ρ= –0.41 at *P*<0.01; Fig. 5; Supplementary Fig. S9A, B at *JXB* online). Twenty-two candidate genes known to be involved in sugar beet bolting and flowering were also studied. Nine of them, displaying the highest variations of normalized values for expression and/or methylation between R and S genotypes, are detailed in Table 2. Four genes had a higher relative expression level in S (*SVP* and the flowering activators *FVE*, *AGL24*, and *CO*), while two genes had a higher relative expression level in R genotypes (the flowering repressor *FLC* and *FUL*). In addition, five genes (*RNMTb*, *FVE*, *FT1*, *FT2*, and *FLC*) had a higher relative methylation level in S genotypes. Using the microarray data collected from sugar beet ESTs and bolting/flowering candidate genes (Table 2), a bolting tolerance model integrating phytohormone signalling genes, response to environment genes, and key bolting/flowering genes that were diferentially expressed and/or methylated between bolting-resistant and bolting-sensitive genotypes after 9 weeks of vernalization is proposed (Fig. 6). The model suggests that upstream regulators of flowering induction have a higher relative methylation level after vernalization in the shoot apical meristem of S genotypes and that their expression levels are in agreement with a potential induction state of flowering in S genotypes and repression state in R genotypes.

**Table 3.** Differentially expressed and differentially methylated genes between bolting-resistant and bolting-sensitive sugar beet genotypes Genes were considered to be differentially expressed between genotypes when the fold change was >2 and the *t*-test *P*-value <0.05, and differentially methylated between genotypes when the fold change was >2.

Sugar beet accession	Sugar beet ID gene	*Arabidopsis thaliana* description	Biological process and/or molecular function	*Arabidopsis* AGI	E-value	Methylation		Expression	
						Methylation level in S^*a*^	Methylation level in R^*a*^	Expression level in S^*a*^	Expression level in R^*a*^
FG345845	Bv_39660_duks.t1	HXXXD-type acyl-transferase-like protein	Suberin and cutin polymer biosynthsesis	AT5G07850	5.00E-39	–0.67	1.16	1.49	-1,62
BQ589458	No significant hit	No significant hit	NA	NA	NA	0.53	–0.52	0.85	-0,66
BQ586261	Bv6_125340_epqo.t1	Germin-like protein 5 (GLP5/ PDGLP1)	Early development, defence, apoptosis	AT1G09560	6.00E-71	0.72	–0.42	0.30	-0,75
FG343208	Bv7u_181680_otyh.t1	Beta-amyrin synthase	Triterpenoid synthesis	AT1G78950	4.00E-74	1.15	–0.04	–0.61	0,93
BQ582907	Bv7_160760_mmcw. t1	Carotenoid epsilon-ring hydroxylase (LUT1/CYP97C1)	Carotenoid biosynthesis, leaf morphogenesis	AT3G53130	5.00E–40	1.22	–0.07	1.01	-0,89
FG344953	Bv3_049870_xxoz.t1	1-Aminocyclopropane-1- carboxylate oxidase-like protein 9	Ethylene biosynthesis	AT1G06620	6.00E-74	1.40	0.17	0.63	-0,41
BQ582428	Bv4u_091120_gpyr.t1	RNA polymerase II transcription mediator activity	Transcriptional regulation of specific signalling pathways	AT3G01435	2.00E-52	1.43	0.21	–1.01	0,88
CF543348	No significant hit	No significant hit	NA	NA	NA	1.59	0.53	–2.36	2,26
CK136737	Bv4_074360_ejeh.t1	Cystatin 6 (CYSB)	Cysteine proteinase inhition, response to stress	AT3G12490	4.00E-35	1.69	0.57	0.99	-0,93
BQ589845	Bv4u_091720_azjm.t1	No significant hit	NA	NA	NA	1.89	0.68	–2.35	2,03
FG345537	Bv9_208140_cjcm.t1	No significant hit	NA	NA	NA	1.99	0.29	–1.01	1,60
BQ489455	Bv7_169260_ewnm. t1	ATPase E1-E2 type family protein	ER to Golgi vesicle-mediated transport	AT1G17500	2.00E-37	2.13	0.63	–2.59	1,44
BQ585496	Bv2_042300_ners.t1	SPX domain-containing protein 4 (SPX4)	Response to phosphate starvation	AT5G15330	8.00E-23	2.48	1.16	0.35	-0,68
BQ488791	No significant hit	No significant hit	NA	NA	NA	2.52	1.43	–0.45	1,03

R, bolting resistant; S, bolting sensitive; NA, not available.

^*a*^Average of the normalized values when several probes corresponded to the same accession.

## Discussion

### Epigenomic approaches in a crop plant

Genomic resources for crop plants such as sugar beet are crucial to elucidate complex physiological processes such as vernalization and bolting. When this study was started, the only public resource for sugar beet genomic sequences was a collection of ~30 000 ESTs in the NCBI dbEST database. However, the sugar beet genome has been released recently, opening up new perspectives (Dohm *et al.*, 2014). EST sequencing only provides data on the coding regions expressed in a given tissue or set of tissues. Therefore, Cot filtration, another reduced-representation sequencing technique without the bias for expressed sequences, was employed to obtain low-copy sequence regions including expressed genes, promoters, introns, and non-expressed genes (Peterson, 2005). In this regard, Cot filtration proved to be an effective tool for sugar beet genome analysis (567 Mbp and 42.3% repetitive sequences; Dohm *et al.*, 2014). The efficiency of Cot filtration as a tool for gene enrichment has been demonstrated in several crop plants (Peterson *et al.*, 2002; Yuan *et al.*, 2003; Lamoureux *et al.*, 2005; Paterson, 2006). In the present study, the sugar beet Cot >940 fraction contained >98% single-copy sequences, confirming the efficacy of the Cot filtration, and led to the assembly of 6231 contigs after sequencing. Only 5% of transcriptomic probes and 11% of methylation probes corresponding to Cot sequences remained after the filtration and normalization steps. Several explanations are possible, such as the low quality of the sequencing and assembly into contigs (1.0% mapped on sugar beet genome), the genetic distance between the genotype and the reference genome, or the quality of the actual version of the reference genome. In addition, fragments from the same gene have probably been re-assembled into several contigs, artificially increasing the number of ORFs used for probe design: 42 000 ORFs were predicted using the data, while only 27 421 protein-coding genes are predicted in the published genome assembly (Dohm *et al.*, 2014). Therefore, a high proportion of predicted ORFs were ‘unreal’ and only 218 ORFs mapped on the genome, representing a very low proportion of potentially ‘efficient’ probes. In addition, 184 ORFs showed sequence homology to genes, but only 84 distinct genes were identified, revealing high redundancy. However, the aim of this study was to expand the sugar beet public database to enable development of the first custom sugar beet expression and methylation microarrays. Thus, 1273 additional filtered probes for expression analysis and 2185 additional filtered probes for methylation analysis were obtained from the sequencing data (Table 1) to complement the public genomic resources. In recent years, microarray technology has demonstrated the power of the high-throughput study of gene expression in unravelling key processes in plant biology (Galbraith, 2006; Clarke and Zhu, 2006). A number of strategies for high-throughput detection of DNA methylation based on microarrays have led to important discoveries (Zhang *et al.*, 2006). In addition, microarrays have become especially relevant for species where little genome information is available and where intensive laboratory work is necessary to gain insight into a particular biological process, as is the case in sugar beet, as well as to identify candidate target genes for future breeding programmes (Martinez-Godoy *et al.*, 2008; Mascarell-Creus *et al.*, 2009). Recently, MeDIP-chip was compared with whole-genome bisulphite sequencing for the detection of DMRs and was also shown to be a reasonable alternative in population epigenetic studies (Wardenaar *et al.*, 2013).

### Bolting tolerance in sugar beet genotypes is associated with the differential expression of an integrative gene network in the shoot apical meristem

The transcriptomic analysis revealed 169 DEGs between R and S vernalized genotypes. These genes are involved in environment perception, hormone signalling, and flowering control. In *Lolium perenne*, 70 cold response genes exhibited variation in expression during vernalization (Ciannamea *et al.*, 2006). Phytohormones, particularly those involved in gibberellin metabolism, are known to be involved in the vernalization response in sugar beet (Mutasa-Göttgens *et al.*, 2009). The present results showed that the expression of genes playing a role in the transition from the vegetative to the floral stage is remodelled in the shoot apical meristem during cold exposure, and in different ways according to the bolting tolerance of genotypes. These gene associations and their expression levels could correspond to a floral determination state in S genotypes, for which a 9 week cold exposure is sufficient to induce bolting under optimal flowering conditions (photoperiod and temperature). The R genotypes could remain in a vegetative state via the control of other genes. Indeed, some flowering activators, such as *BvFVE*, *BvAGL24*, and *BvCOL1*, had a higher relative expression level in S genotypes, while the floral repressor *BvFL1* had a higher relative expression level in R genotypes, corresponding to a suitable context for the bolting initiation. In a previous study, R and S genotypes also exhibited different expression patterns for key regulators of the vernalization in sugar beet (Trap-Gentil *et al.*, 2011; Hébrard *et al.*, 2013). Recently, the *BvFL1* gene was functionally characterized using RNAi and overexpression in transgenic sugar beet plants (Vogt *et al.*, 2014). *BvFL1* RNAi neither eliminated the requirement for vernalization of biennial beets nor had a major effect on bolting time after vernalization, while overexpression of *BvFL1* resulted in a moderate late-bolting phenotype. Neither down-regulation of *BvFL1* by RNAi nor its overexpression affected the expression of the central floral regulators *BvBTC1*, *BvFT1*, and *BvFT2* in a consistent way. These data demonstrated that *BvFL1* is not a major regulator of the vernalization response in sugar beet and its role has to be clarified. In the present study, *BvFT1* and *BvFT2* were not retained after the filtration of the transcriptomic probes, indicating no expression or a low expression level in the shoot apical meristem, in agreement with the findings of Pin *et al.* (2010). In the same way, *BvRNMTa* and *BvRNMTb* seemed not to be expressed after 9 weeks of vernalization, in agreement with previous data (Hébrard *et al.*, 2013). In 2012, Mutasa-Göttgens *et al*. generated a sugar beet transcriptome by RNA sequencing (RNA-seq) and compared the expression level of genes in different genotypes before and after 21 weeks of vernalization and with or without gibberellin treatment. Some genes highlighted in that study were also evidenced in the present study, such as *BQ584677* corresponding to the *locus 6819* in Mutasa-Göttgens *et al.* (2012). This EST has strong homology with the *FUL* gene of *Arabidopsis*, which is involved in floral meristem identity, and was expressed differentially between R and S sugar beet genotypes. This RNA-seq analysis also revealed 20% of genes that were differentially expressed between genotypes with distinct vernalization requirements. These results indicated that such sugar beet genotypes are both genetically and transcriptionally distinct, in agreement with the present data. Similar divergences have been observed between Nipponbare and indica rice varieties, which revealed 1525 DEGs (Chodavarapu *et al.*, 2012).

### Bolting tolerance in sugar beet is associated with the hypermethylation of an integrative gene network in the shoot apical meristem of sensitive genotypes

The methylation analysis revealed 111 DMRs between R and S vernalized genotypes. Therefore, microarray is an efficient tool to identify DMRs at a genomic scale using available public genomic resources. In a previous study, 39 DMRs between genotypes were identified during vernalization by the use of the restriction landmark genome scanning (RLGS) method (Hébrard *et al.*, 2013). Here, the 111 DMRs all had a higher relative methylation level in S genotypes compared with the R genotypes. These results are in agreement with previous global DNA methylation data revealing the hypermethylated status of bolting-sensitive sugar beet genotypes after 9 weeks of vernalization (Trap-Gentil *et al.*, 2011). These elements indicated that R and S genotypes have distinct DNA methylation patterns during vernalization, allowing their early discrimination. DNA methylation divergences in the same species have already been reported in two *Arabidopsis* ecotypes, Landsberg *erecta* and C24, and their reciprocal hybrids (Shen *et al.*, 2012). In rice, the Nipponbare and indica varieties revealed 7% of epimutations (Chodavarapu *et al.*, 2012). The opposite DNA methylation and expression patterns observed in sugar beet genotypes during vernalization may reflect two meristematic determination states, one corresponding to a vegetative form and the other to a floral form. Indeed, in *Azalea japonica*, floral buds have been shown to be hypermethylated compared with vegetative buds (Meijon *et al.*, 2009).

Fourteen sequences that were both differentially expressed and differentially methylated (DEMSs) between R and S genotypes were also identified. In the same way, 45 DEMSs have already been reported between the Nipponbare and indica rice varieties (Chodavarapu *et al.*, 2012). Expression and DNA methylation of these 14 sugar beet DEMSs were negatively correlated, in agreement with other studies in *Arabidopsis* (Zhang *et al.*, 2006; Lister *et al.*, 2008), poplar (Vining *et al.*, 2012), and rice (He *et al.*, 2010). The biological significance of gene-body methylation is still not clear, although it has been proposed that it may suppress aberrant transcription from cryptic promoters inside the genes or participate in splicing variants (Zilberman *et al.*, 2007; Chodavarapu *et al.*, 2010; Lauria and Rossi, 2011). Nine of these 14 DEMSs exhibited putative functional annotations. Two sequences, encoding *CYSTATIN 6* (*CYSB*, FG345694) and a potential *RNA POLYMERASE II TRANSCRIPTION MEDIATOR* subunit (BQ582428), have already been shown to be involved in plant development and response to environmental stress. Cystatins are a group of proteins that are widespread in eukaryotes and inhibit cysteine proteases by direct interaction with the active site (Turk and Bode, 1991; Margis *et al.*, 1998). In plants, it has been suggested that cystatins may play a role in storage proteins, programmed cell death, defence mechanisms, regulation of endogenous proteases, and catabolism, germination, and seed maturation (Massonneau *et al.*, 2005). In *Arabidopsis*, seven *CYS* genes (*AtCYS1*–*AtCYS7*) have been identified (Martinez *et al.* 2005). The expression of *AtCYSa* and *AtCYSb* (synonymous with *AtCYS3* and *AtCYS6*, respectively) is strongly induced in cells and seedlings by multiple abiotic stresses including high salinity, drought, oxidative stress, and cold; overexpression of these genes in transgenic yeast and *Arabidopsis* plants increases resistance to these stresses (Zhang *et al.*, 2008). Other studies have reported an up-regulation of cystatin mRNA transcripts in organs submitted to adverse growth conditions, including low temperatures in amaranth, barley, maize, or wheat (Valdés-Rodriguez *et al.*, 2007; Gaddour *et al.*, 2001; Massonneau *et al.*, 2005, Christova *et al.*, 2006, respectively).

The Mediator complex is a large multiprotein complex conserved in all eukaryotes which acts as a bridge between the RNA polymerase II complex and transcription factors (Kim *et al.*, 1994; Koleske and Young, 1994). Developmental aberrations have been reported for *Arabidopsis* mutants in a number of genes subsequently identified as encoding Mediator subunits. For example, the loss of *SENSITIVE-TO-FREEZING6* (*SFR6*/*MED16*) function renders mutants unable to cold-acclimate and disrupts transcriptional outputs without low-temperature gene regulation, affecting expression of the flowering time pathway and circadian clock genes (Knight *et al.*, 2008). The *struwwelpeter* (*swp*/*med14*) mutants exhibited reduced cell numbers in all aerial organs, changes in the window of cell proliferation, and perturbations in the shoot apical meristem (Autran *et al.*, 2002). Recently, Zheng *et al.* (2013) showed that *Arabidopsis* MEDIATOR SUBUNIT 18 (MED18) affects flowering time and floral organ formation through *FLC* and *AG*. In the *MED18* loss-of-function mutant, flowering time was delayed and floral organ number as well as *FLC* and *AG* mRNA levels were altered. *FG345694* and *BQ582428* constitute interesting candidate genes for bolting tolerance.

Regarding another seven annotated sequences (*FG345845*, *FG343208*, *BQ582907*, *FG344953*, *BQ489455*, *BQ586261*, and *BQ585496*), no relationship to bolting has been established according to the literature, and further functional studies are needed to show their putative involvement in this physiological process. The possible role of these 14 DEMSs in bolting tolerance has yet to be defined. The study of corresponding *Arabidopsis* mutants, as in Hébrard *et al.* (2013), or RNAi sugar beet mutants (Pin *et al.*, 2010, 2012; Vogt *et al.*, 2014) will be relevant to elucidate their implication.

The bolting tolerance model (Fig. 6) is in agreement with phenotypic plasticity resulting from the control of plasticity genes modulated by environmental conditions (DNA methylation and gene expression) on the activity of organogenesis genes. The model suggests that sugar beet bolting tolerance corresponds to a regulation pathway whose genes are activated precociously in bolting-sensitive genotypes (through DNA methylation and gene expression variations) compared with the bolting-resistant genotypes. To test this model, methylome and transcriptomic analyses (using whole-genome bisulphite sequencing and RNA-seq, respectively) could be performed using the sugar beet genome that is now released (Dohm *et al.*, 2014). This study could be applied on a larger set of genotypes vernalized or not during short cold periods (from 1 to 3 weeks) in order to identify the early mechanisms controlling bolting tolerance.

### The sugar beet mitochondrial genome is affected by transcriptomic and methylation changes after vernalization related to bolting tolerance

The sugar beet mitochondrial genome was analysed for gene expression and DNA methylation in both R and S genotypes after 9 weeks of vernalization. The six genotypes had the same haplotype (ON type). Only two probes corresponding to *ORF104* and *COX2* were differentially expressed between R and S genotypes. Surprisingly, 3211 methylation probes were retained after the filtration step, and a mitochondrial methylome was established for the two types of genotypes. The methylation normalized values obtained were negative and very low for both groups of genotypes, indicating a very low methylation level. Methylation has already been detected in the mitochondrial DNA of several organisms, such as human, mouse, hamster, and a number of plant species (Nass, 1973; Shmookler Reis and Goldstein, 1983; Pollack *et al.*, 1984; Simkova, 1998; Bellizzi *et al.*, 2013). Other studies suggested that DNA methyltransferases could have access to different sites on the mitochondrial DNA, depending on the levels of protein occupancy (Rebelo *et al.*, 2009) and could be translocated into the mitochondria (Shock *et al.*, 2011). The sugar beet mitochondrial genome had a higher relative methylation level in R genotypes compared with the S genotypes, in contrast to the nuclear DNA. In *Sequoia sempervirens*, mitochondrial methylation is higher in juvenile shoots compared with adult shoots (6.6% versus 7.8–8.2%, respectively) and vice versa for the nuclear methylation (8% versus 6.5–7.5%, respectively; Huang *et al.*, 2012). The DNA methylation machinery could be regulated in different ways in the mitochondrion and in the nucleus, and according to the developmental stages. In addition, 510 differentially methylated probes have been identified between R and S genotypes, and some of them were clustered in two loci of 10–15kb. In these loci, only three ORFs were expressed (*ORF152*, *ORF102b*, and *ORF192*), and their expression level was higher in S compared with the R genotypes. This result suggests that the increased methylation level in these loci could reduce the expression of the corresponding coding sequences in R genotypes. This relationship has been demonstrated for some *Arabidopsis* nuclear genes (Zhang *et al.*, 2006; Lister *et al.*, 2008). Further analyses are needed to understand the role of these two mitochondrial loci that could be good candidates to distinguish S and R genotypes by assisted marker selection.

### Conclusion

Flowering is regulated by seasonal cues to help ensure reproductive success, synchronous flowering in agriculture, and maximal seed yield. Vernalization is the best known example of an environmentally induced epigenetic switch (Song *et al.*, 2012). Recently, using epigenetic recombinant inbred lines (*epiRILs*), DMRs have been shown to act as epigenetic quantitative trait loci (*QTLepi*), accounting for 60% of the heritability for flowering time in *Arabidopsis* (Cortijo *et al.*, 2014). However, the genotypic tolerance to bolting and flowering, particularly important for breeders, is still poorly understood in many crops. In *Arabidopsis*, quantitative modulation of chromatin silencing mechanisms through *cis* polymorphisms explains natural variation in vernalization (Coustham *et al.*, 2012). The present results showed that bolting tolerance is associated with a genotype-dependent remodelling of DNA methylation and expression of an integrative gene network involved in environmental perception, hormone signalling, and flowering in the shoot apical meristem. One perspective will be to construct the clusters of each bolting-tolerant group by development and use of new SNPs discovered in the DEG/DMR genes. In addition, the shoot apical meristem is a particularly relevant tissue in which to study the role of epigenetics in plant phenotypic plasticity and adaptation, and seems to be the site of a specific epigenetic control (Baubec *et al.*, 2014). Taken together, the present data, in agreement with previously published studies on sugar beet (Pin *et al.*, 2010; Trap-Gentil *et al.*, 2011; Hébrard *et al.*, 2013), open up new perspectives for sugar beet breeding. Moreover, a recent study revealed for the first time a major QTL for bolting tolerance in sugar beet, representing a major contribution towards the development of a winter sugar beet with controlled bolting behaviour (Pfeiffer *et al.*, 2014). The complete genomic sequence of sugar beet has been recently published (Dohm *et al.*, 2014) and will support molecular breeding, especially by the development of genome-wide genetic and epigenetic markers for relevant agronomic traits.

## Supplementary data

Supplementary data are available at *JXB* online.

Figure S1. A Cot curve, a graphic representation of a genome.

Figure S2. Diagram representing the strategy used to identify candidate genes for bolting tolerance.

Figure S3. Histogram representing the read length distribution after 454 high throughput sequencing.

Figure S4. TAIR percentage of gene ontology (GO) terms for ‘Biological Process’ and ‘Molecular Function’ of annotated sugar beet ORFs from the Cot fraction.

Figure S5. Box plots showing the distribution of the 22 448 transcriptomic filtered probes after the normalization step for the six sugar beet genotypes.

Figure S6. Fold change expression using qRT–PCR validates transcriptomic microarray data.

Figure S7. Histogram showing the distribution of the 19 574 methylation-filtered probes after the normalization step for the six sugar beet genotypes.

Figure S8. Bisulphite sequencing confirmation of DNA methylation changes detected with the methylation microarray.

Figure S9. Relationship between methylation and expression in sugar beet genotypes after 9 weeks of vernalization treatment.

Table S1. Sequence of primers.

Table S2. Assembly data summary.

Table S3. Functional analysis of the Cot fraction.

Table S4. Contigs with a blast hit in the sugar beet genome.

Table S5. ORF annotations.

Table S6. ORFs with a blast hit in the sugar beet genome.

Table S7. 169 differentially expressed genes (DEGs).

Table S8. 111 differentially methylated regions (DMRs).

Supplementary Data

## Abbreviations:

BD: bolting delay
BI: bolting index
CH3: methylation
Cy: cyanine
DEG: differentially expressed gene
DEMS: differentially expressed and differentially methylated sequence
DMR: differentially methylated region
EST: expressed sequence tag
FL1: *FLOWERING LOCUS C*-like
FT: *FLOWERING LOCUS T*
HAP: hydroxyapatite
ORF: open reading frame
QTL: quantitative trait locus
R: bolting resistant
RLGS: restriction landmark genome scanning
S.: bolting sensitive.
