# Supplementary material for: Epigenomics and bolting tolerance in sugar beet genotypes
Source: J Exp Bot. 2015 Oct 13;67(1):207–25. doi: 10.1093/jxb/erv449 (PMC4682430; doi:10.1093/jxb/erv449)
Supplement: Supplementary Data [file supp_67_1_207__index.html]

Epigenomics and bolting tolerance in sugar beet genotypes — Epigenomics and bolting tolerance in sugar beet genotypes — Supplementary Data 

# Epigenomics and bolting tolerance in sugar beet genotypes

## Supplementary Data

Data files

- Supplementary Data - Supplementary Data
- Supplementary Data - Supplementary Data
- Supplementary Data - Supplementary Data
- Supplementary Data - Supplementary Data
- Supplementary Data - Supplementary Data
- Supplementary Data - Supplementary Data
