# Supplementary material for: Epigenomics and bolting tolerance in sugar beet genotypes
Source: J Exp Bot. 2015 Oct 13;67(1):207–25. doi: 10.1093/jxb/erv449 (PMC4682430; doi:10.1093/jxb/erv449)
Supplement: Supplementary Data [file supp_erv449_Supplementary_data.pdf]

Journal of Experimental Botany

Epigenomics and bolting tolerance in sugar beet genotypes

Claire Hébrard, Daniel G Peterson, Glenda Willems, Alain Delaunay, Béline Jesson, Marc Lefebvre, Steve Barnes and Stéphane Maury

Supplementary data

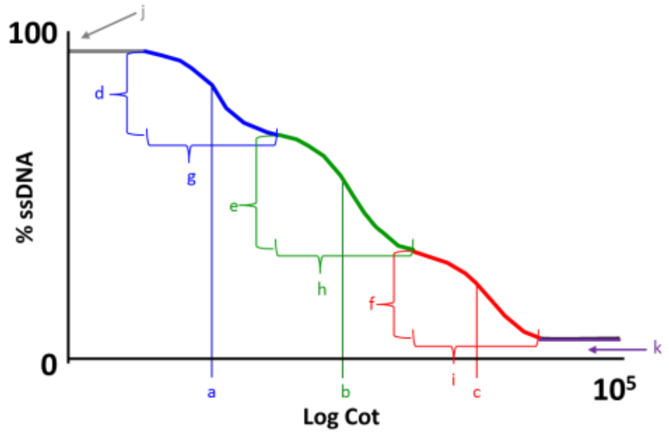

Supplementary Figure S1: A Cot curve, a graphic representation of a genome. In this example, the curve consists of three components; a highly repetitive (HR - red) component, a moderately repetitive (MR - green) component, and a single/low-copy (SL - blue) component characterized by fast, intermediate, and slow reassociation, respectively. Each component has a Cot½ value which marks the point at which 50% of the DNA sequences in that component have reassociated (a, b, and c). The height of each component (d, e, and f) is reflective of the fraction of the genome contained in that component. 80% of the DNA in a component reassociates between 0.1*y* and 10*y* (g, h, and i) where *y* is the component's Cot½. Even at Cot values approaching zero the percentage of unreassociated DNA is always less than 100% due to intramolecular foldback (j). Additionally, some sequences are damaged (presumably during shearing) and will not reassociate (k).

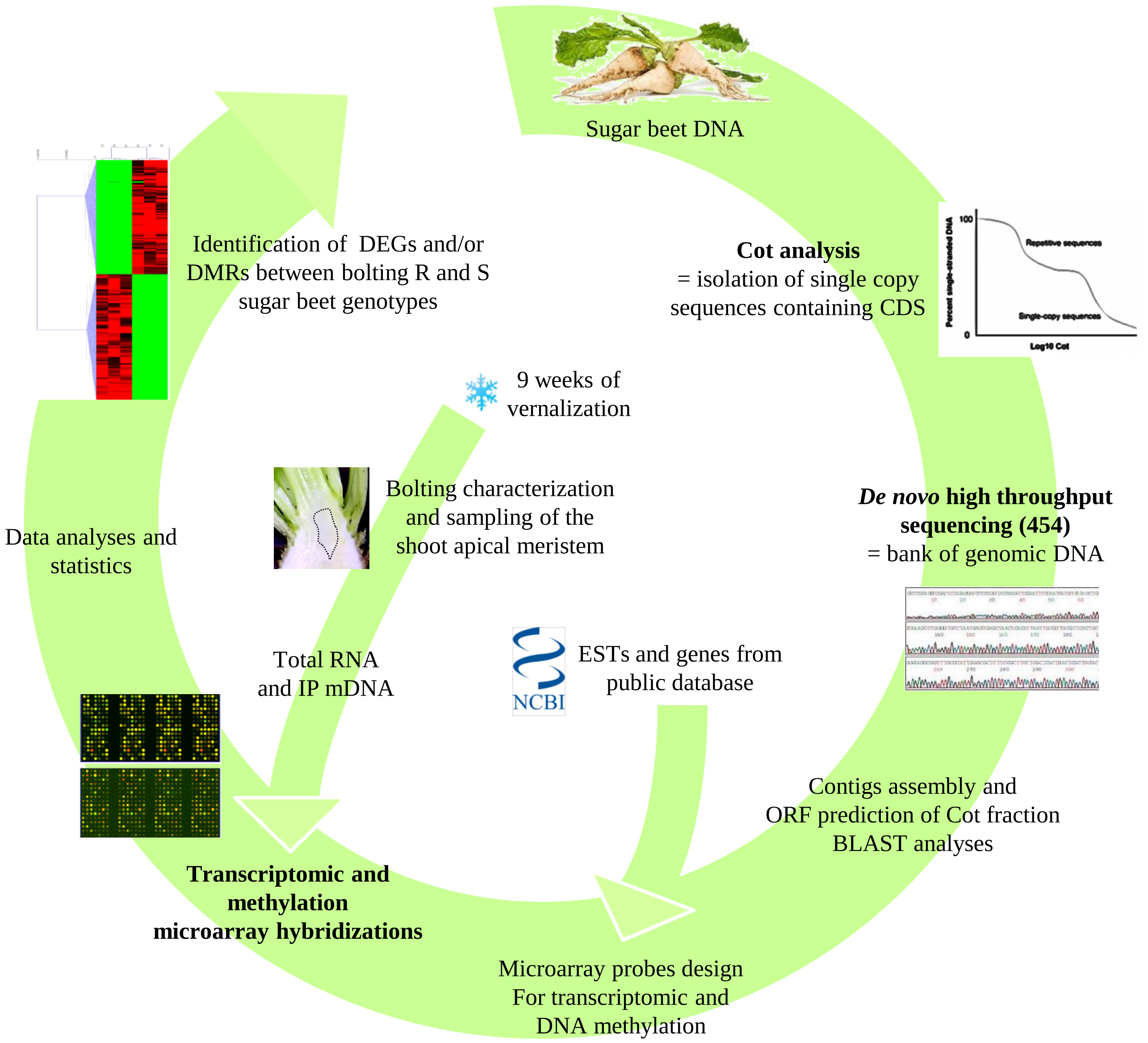

Supplementary Figure S2: Diagram representing the used strategy to identify candidate genes for bolting tolerance. IP mDNA: immuno-precipitated methylated DNA; DEG: differentially expressed gene; DMR: differentially methylated region; R: bolting resistant; S: bolting sensitive.

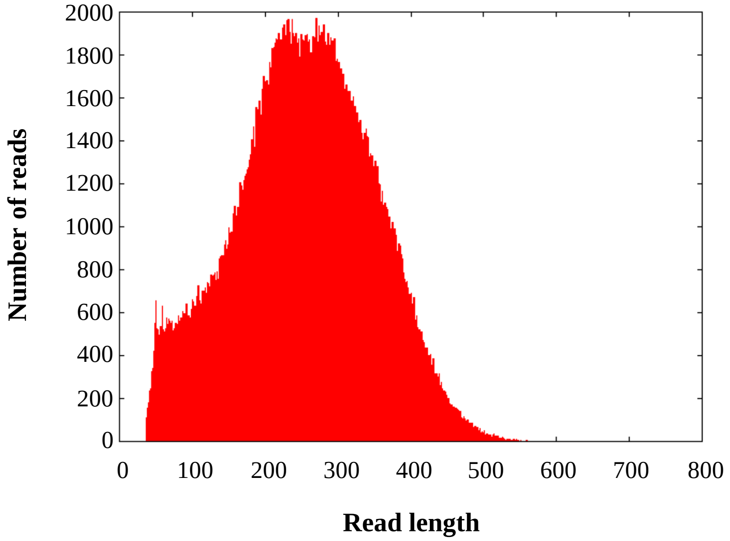

Supplementary Figure S3: Histogram representing the read length distribution after 454 high throughput sequencing.

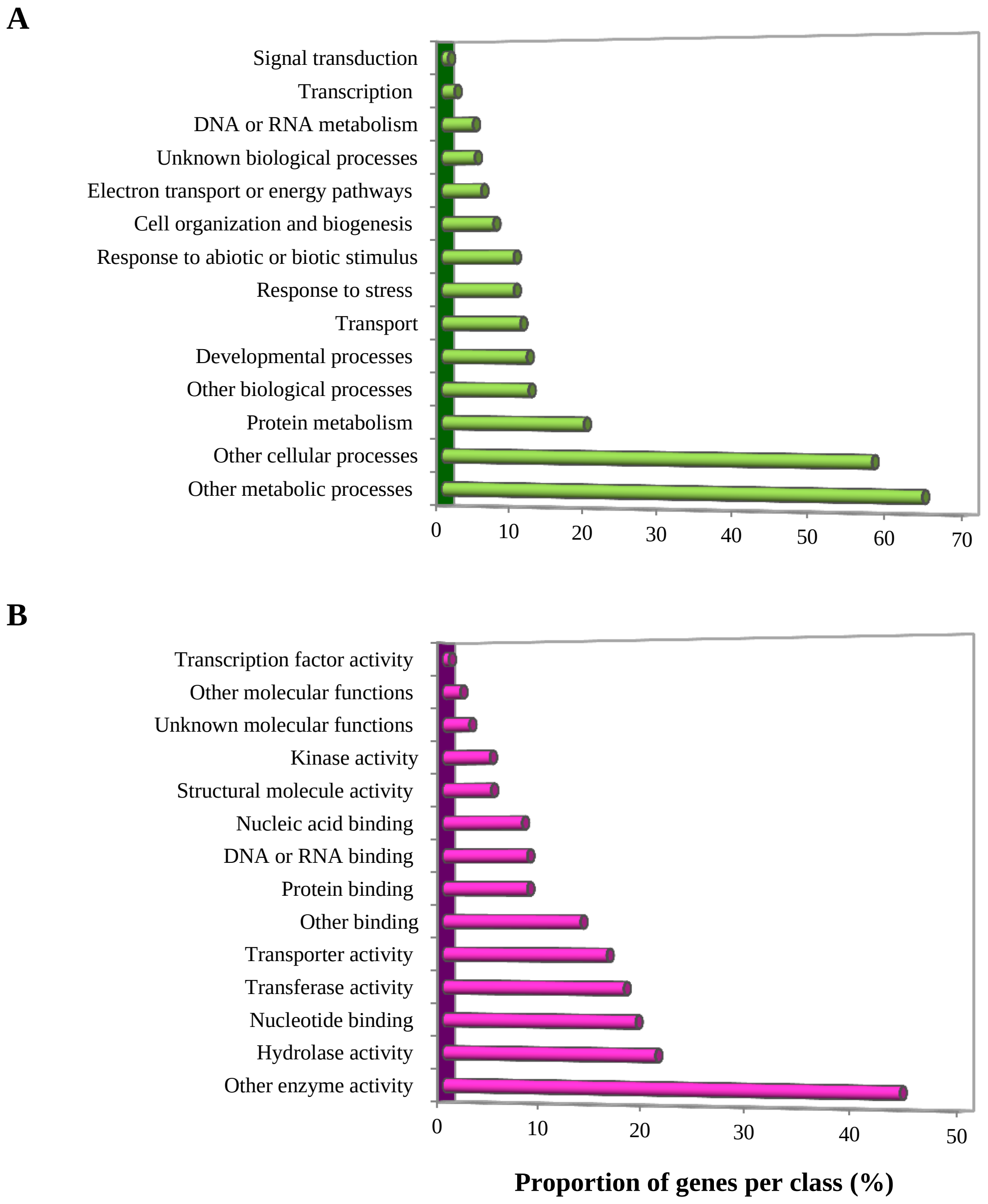

Supplementary Figure S4: TAIR percent of gene ontology (GO) terms for (A) "Biological Process" and (B) "Molecular Function" of annotated sugar beet ORFs from C_0t fraction.

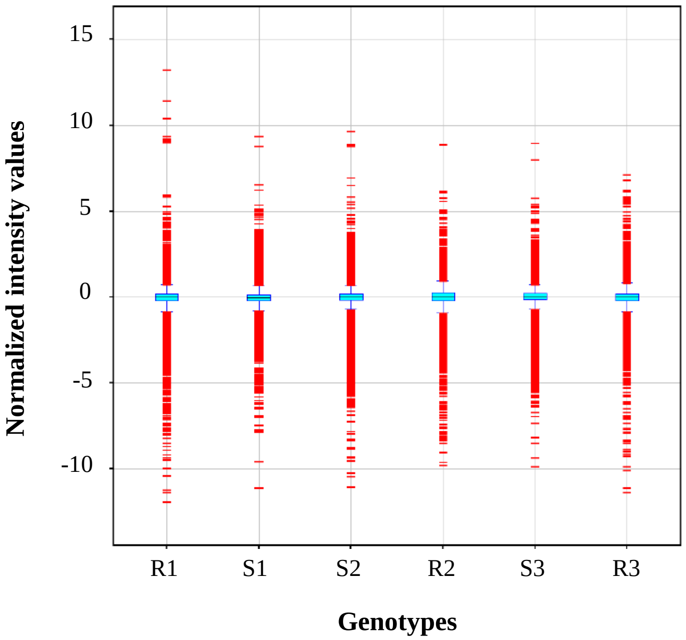

Supplementary Figure S5: Box plots showing the distribution of the 22 448 transcriptomic filtered probes after normalization step for the three bolting resistant (R1 to R3) and the three bolting sensitive (S1 to S3) sugar beet genotypes.

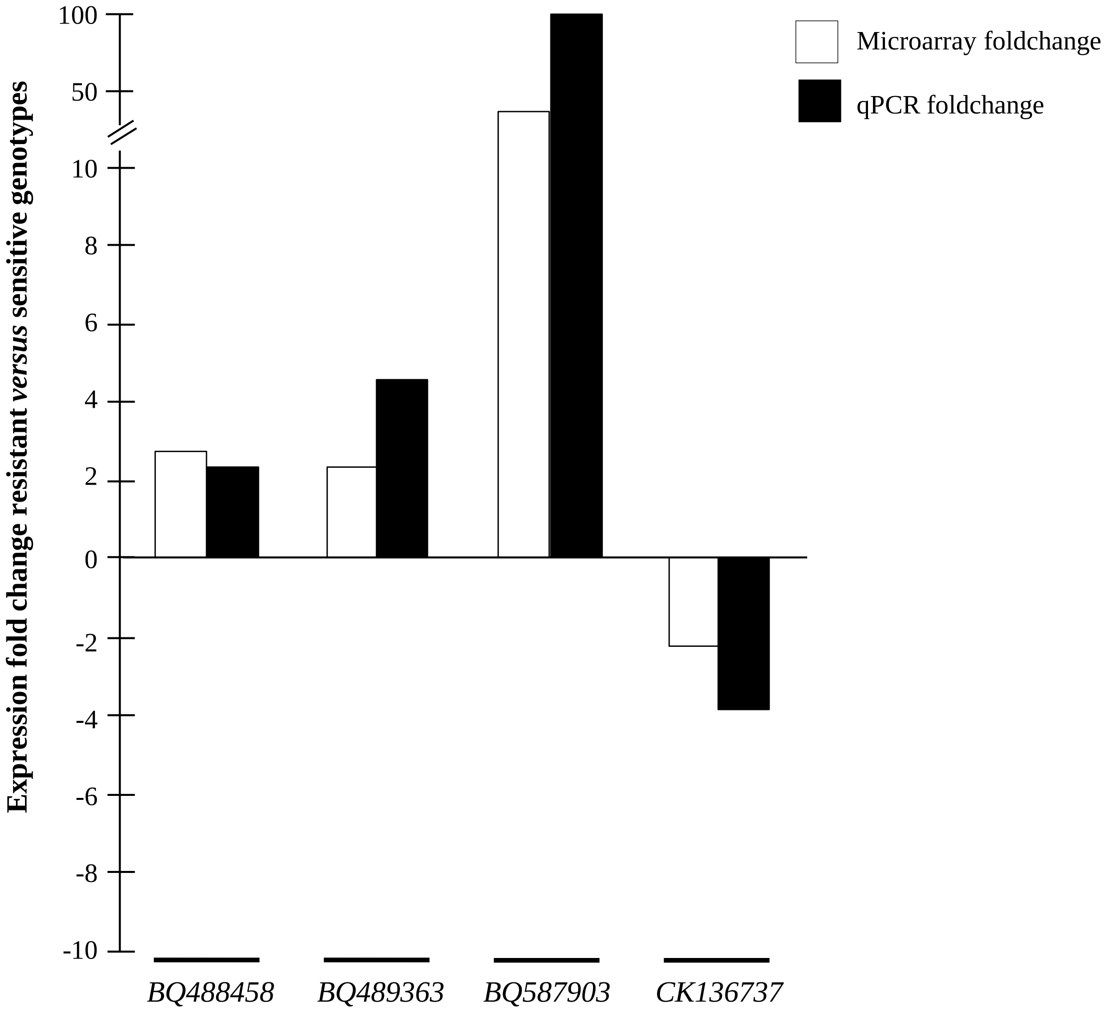

Supplementary Figure S6: Fold-change expression using qRT-PCR validates transcriptomic microarray data. Four genes showing significant expression variations in our transcriptomic microarray data (white bar) were analyzed by qRT-PCR (black bar).

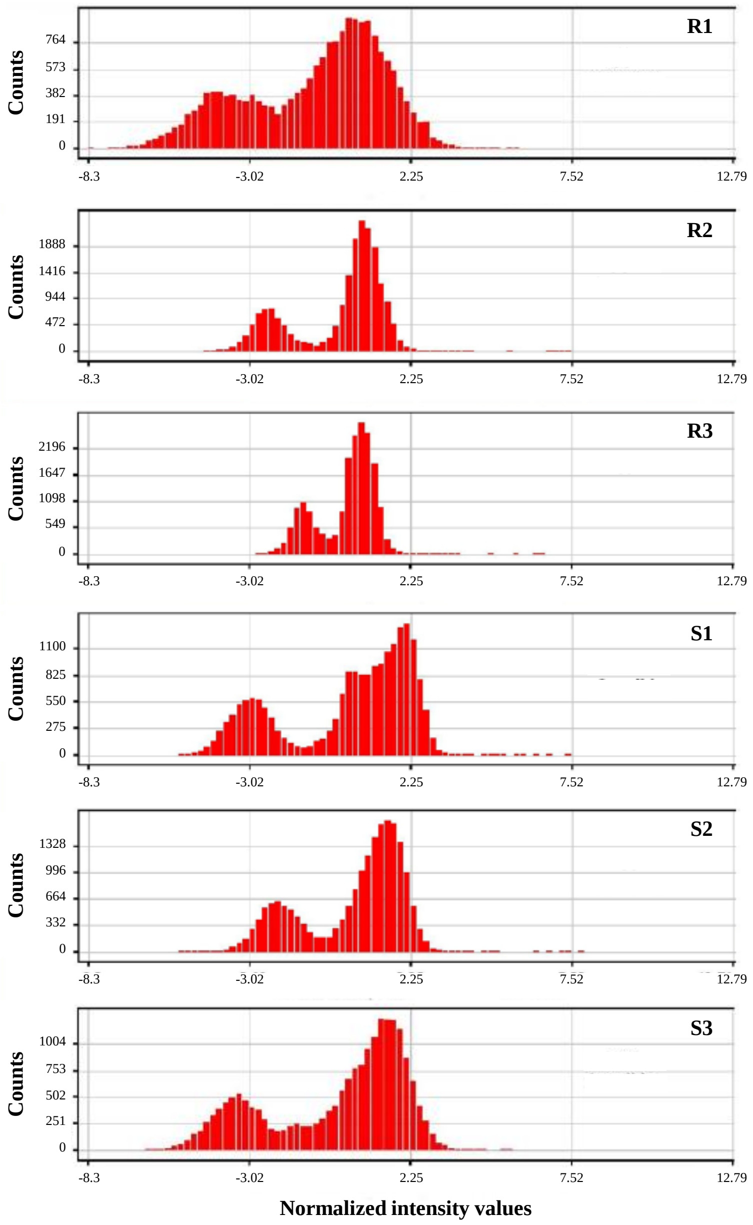

Supplementary Figure S7: Histogram showing the distribution of the 19 574 methylation filtered probes after normalization step for the three bolting resistant (R1 to R3) and the three bolting sensitive (S1 to S3) sugar beet genotypes.

Bisulfite sequencing results **Microarray results**

BI543770

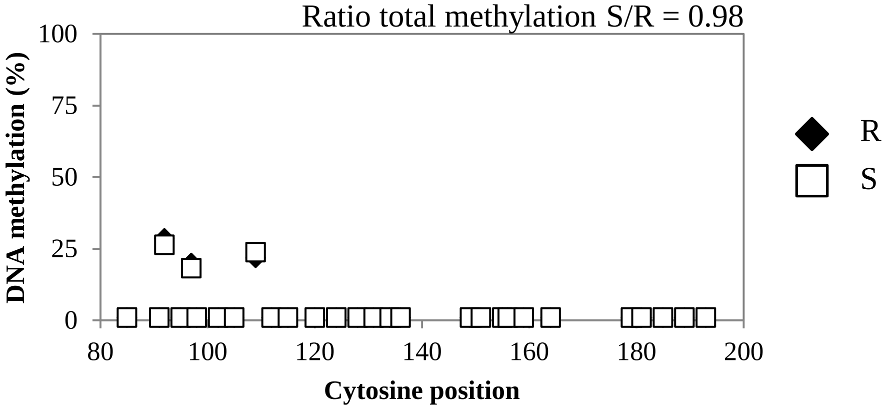

Very low methylation signal with low change S versus R (FC=0.97)

BQ593527

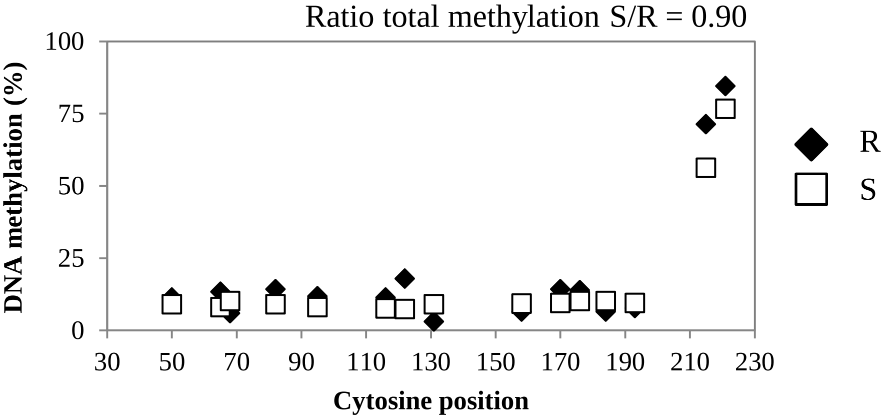

Low methylation signal with low change S versus R (FC=0.96)

HM448911

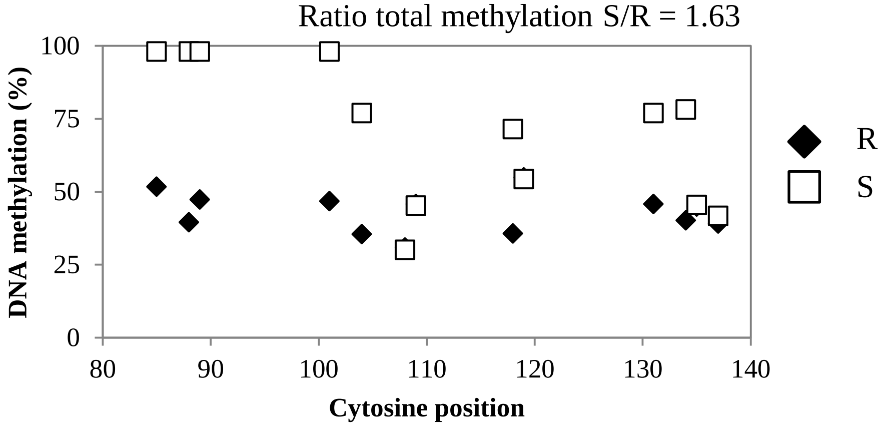

High methylation signal with hypermethylation in S versus R (FC=1.70)

Supplementary Figure S8: Bisulfite sequencing confirmation of DNA methylation changes detected with the methylation microarray. DNA methylation profiles for bolting resistant (R, black diamonds) and sensitive genotypes (S, white squares) are given for three different genes: *BI543770*, *BQ593527* and *HM448911*. Each dot represents a cytosine. For each locus, the right panel indicates the nature of DNA methylation changes between conditions as detected by the methylation microarray.

A

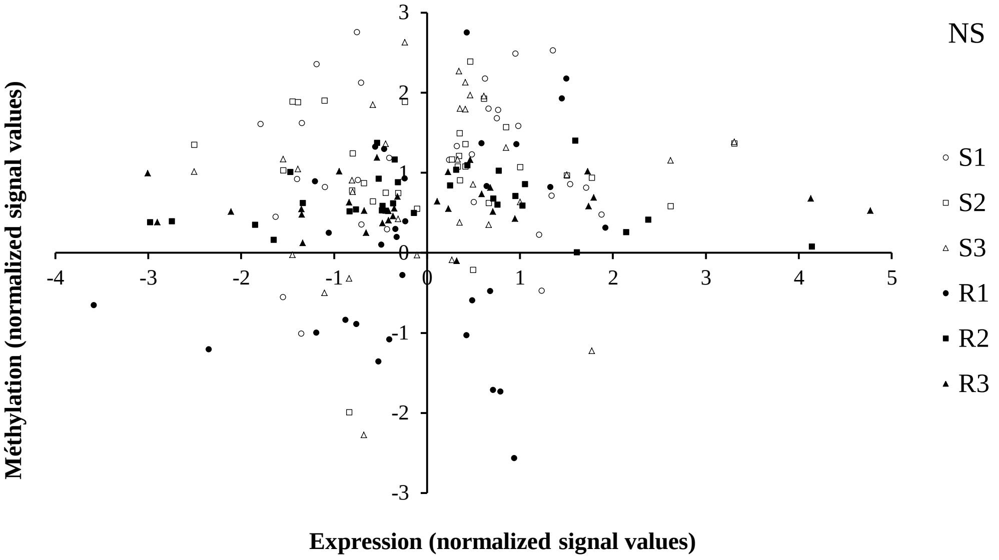

B

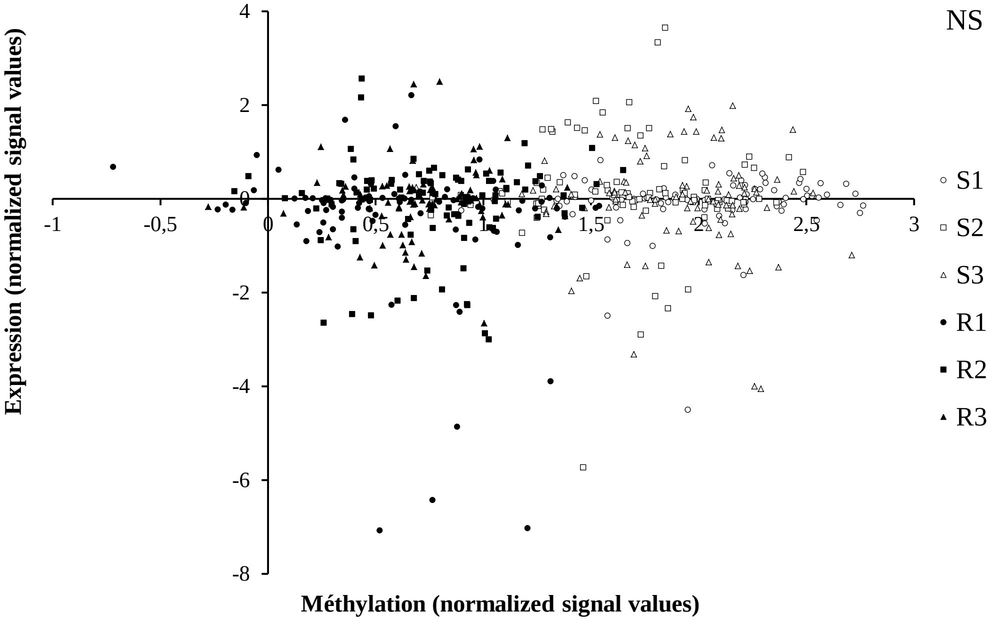

Supplementary Figure S9: Relation between methylation and expression in sugar beet genotypes after 9 weeks of vernalization treatment. White circles, squares and triangles represent the 3 bolting sensitive genotypes while black circles, squares and triangles correspond to the bolting resistant genotypes. (A) Methylation level vs expression level for the 169 differentially expressed genes (when the corresponding methylation probes are retained after filtration). (B) Expression level vs methylation level for the 111 differentially methylated regions (when the corresponding expression probes are retained after filtration).

Supplementary Table S1: Sequence of primers. For degenerated primers, Y = C, T and R = G, A.

**Experiment**	**Sequence ID**	**Primer sequence**
Bisulfite sequencing	BI543770	F 5' ATAGGGAAATGGTYTGAYAGATG 3'<br>R 5' AAARCCTTTACATCARTCCAA 3'
	BQ488791	F 5' AGYTTAGTTTGYTAGTGGTGG 3'<br>R 5' TRTCCCCARARAAAAACAATA 3'
	BQ587382	F 5' GGYGGAGGGGAAGATGTC 3'<br>R 5' CCARATCATCATCAAAARCC 3'
	BQ593527	F 5' AGAAGAGGAAGAGGAATTTAGAAG 3'<br>R 5' CCAAATAATCCRCAATARAATC 3'
	CV301724	F 5' AGGGAATGYTAAGYYGTTATA 3'<br>R 5' CTTCRACARATTRTCCCAAAT 3'
	BvFT2	F 5' TTGTTTGGTTGTATATAGGAAGT 3'<br>R 5' CTTTTRAACTCATCATATTCTTC 3'
	FG345537	F 5' ATTTGTAAAGGAYGTGAGAGAT 3'<br>R 5' ATAATCCAACATTATCATCATCA 3'
Quantitative PCR	*BvUBIQUITIN*	F 5' TCGAAGATGGCCGTACTTTGGC 3'<br>R 5' CCCTCAAACGGAGAACCAAGTG 3'
	BvATP SYNTHASE BETA SUBUNIT	F 5' TCATCGGTGCCGTTGTTGATGTC 3'<br>R 5' ATCAAGCACCTCAAGGGCAGTC 3'
	BQ488458	F 5' ACGGTCAGCAGAGAACTCATGG 3'<br>R 5' ATCAATCTCAGCCGCACGATTAGC 3'
	BQ489363	F 5' ATCTCGCCCACCCAATTTCCAG 3'<br>R 5' GAAAGTTGGGCACGGCAGATCAAC 3'
	BQ587903	F 5' ATGGAACATGGTTTGCGCTTGG 3'<br>R 5' ACACCCTTCCTAACAGCACCAC 3'
	CK136737	F 5' AGTATGTGGGTGATGCCGATGC 3'<br>R 5' GTACGGGTCATCAGTAGACCTAGC 3'

Supplementary Table S2: Assembly data summary.

Category	Quantity
Total number of reads	466 386
Total number of bases	117 949 495
Number of aligned reads	73110
Number of aligned bases	15 608 909
Number of assembled reads	40 645
Number of partial reads	32 296
Number of singleton reads	364 748
Number of repeat reads	292
Number of outliers reads	24 683
**Large contigs (>500 nt)**	
Number of contigs	2 211
Number of bases	1 841 482
Average contig size	832
N50 contig size	781
Largest contig size	14 860
**All contigs**	
Number of contigs	6 231
Number of bases	2 953 454

Supplementary Table S3: Functional analysis of the Cot fraction.

Class	Number of elements	Length (bp)	%
**Repeat sequences**		**40 952**	**1,39**
Retroelements	14	2 784	
DNA transposons	3	145	
Unclassified	3	1 035	
Small RNA	37	13 503	
Simple repeats	238	13 336	
Low complexity	265	10 149	
**Single copy sequences**		**2 912 502**	**98,61**
Contigs	6 231		
Potential ORFs predicted from contigs	42 004		
Non-redondant annotations between species	1629		
